# Experimental study on anchorage performance of rockbolts by adding steel aggregates into resin anchoring agents

**DOI:** 10.1371/journal.pone.0255046

**Published:** 2021-07-28

**Authors:** Ming Zhang, Jun Han, Chen Cao, Shuangwen Ma

**Affiliations:** 1 College of Mining, Liaoning Technical University, Fuxin, China; 2 Liaoning Province Coal Resources Safety Mining and Clean Utilization Engineering Research Center, Fuxin, China; 3 EIS, CME, University of Wollongong, Wollongong, NSW, Australia; China University of Mining and Technology, CHINA

## Abstract

Pull-out testing was carried out to evaluate the effects of shape, size and concentration of steel aggregates on anchorage performance. Steel grit with particle sizes of 1.5, 2.0, and 2.8 mm and steel shot diameters of 1.4, 2.0, and 2.5 mm were used as steel aggregates and were added into the resin anchoring agent. For each kind of steel aggregate, either 30, 40 or 50 aggregates were used to evaluate the effects of different steel aggregate densities. Anchorage specimens were prepared using ϕ20mm rebar bolts and steel sleeves. Compressive and shear strengths of resin containing steel aggregates, the pullout curve, and the circumferential strain of the sleeves were measured, and the energy consumption was calculated. Results show that compressive and shear strengths of resin containing steel grit and steel shot are increased by 8.4%-17.0% compared to pure resin. For the aggregate numbers of 30, 40 and 50, the anchoring force is increased by 7.9%, 7.5% and 6.5%; energy consumption is increased by 19.2%, 15.0% and 18.6%; and the circumferential strain of the specimen is increased by 28.4%, 25.1% and 39.5%, respectively. The effect of aggregate size on anchoring performance is significant; that is, the aggregate sizes of 1.4~1.5, 2.0 and 2.5~2.8 mm increase the anchoring force, energy consumption and sleeve circumferential strain by 8.5%, 4.6% and 8.7%, 16.0%, 8.4% and 28.4%, and 17.9%, 23.3% and 51.9%, respectively. The relationships of the anchoring force, energy consumption, and circumferential strain with steel aggregate quantity and size are formulated. Results show that the addition of steel aggregates increases the compressive and shear strengths of the resin, and steel aggregate quantity and size have significant impact on anchoring performance. This paper provides the basis for optimization of resin anchoring agents used in the mining industry. The impact of anchoring agent shear strength and residual shear strength on the anchoring effect were also discussed based on the failure analysis of the anchoring section.

## Introduction

Resin anchoring agents are bonding agents composed of unsaturated polyester resins, curing agents, accelerators and other auxiliary materials. They are widely used in building reinforcement, wellbore installation, highway repair, tunnel construction, foundation rooting, equipment foundation, hydropower prestressing, roadway support, anchor reinforcement and component anchorage [1–9]. For example, encapsulated resin anchoring agents have been increasingly used in rock bolting for strata stabilization around underground excavations in the coal mining industry for the past three decades. It is estimated that over 300 million bolts installed in coal mines worldwide employ polyester bolt resins. If converted to a 330 mm long cartridge, they can encircle the Earth at the equator approximately seven times each year [2].

A fully grouted anchor is classified as a Continuous Mechanically Coupled (CMC) system comprised of a tendon that is inserted into a drilled hole and then grouted. The main function of the grout, which fully fills the annulus between the rock bolt and the borehole wall, is to provide a mechanism for load transfer between the rock mass and the reinforcing element [10–15]. At present, it is commonly accepted that higher bond strengths can be realized with 4–6 mm resin annulus [16–19], and important factors influencing the quality of encapsulation in rock bolting were reported as borehole diameter, resin annulus thickness, installation time, gloving, and hole over drill [16]. It should be noted that the effectiveness of the installed roof bolts can be compromised by gloving. Gloving refers to the plastic cartridge of a resin capsule encasing a length of the bolt, typically with a combination of mixed and unmixed resin filler and catalyst remaining within the cartridge. Gloved and unmixed portions reduce the effective anchor length and adversely affect reinforcement [11,12,20], and this can be alleviated by adding steel aggregates into the resin [21].

Studies have shown that adding aggregates into grouting materials has a noticeable effect on their mechanical properties and anchoring performance. For instance, Xu et al [22] found that the compressive strength of concrete can be improved by adding coarse aggregates with appropriate size, and concluded that increasing aggregate interlock effects could improve penetration resistance and volume stability. Miah et al [23] found that adding steel slag aggregates could significantly improve compressive strength. Dalhat [24] found that adding less than 20% aggregates can improve the compressive and flexural strengths of the low epoxy-resin mortar, and the improvement in flexural properties are due to the additional mechanical bending support offered by the aggregates through surface bonding with the sand-resin matrix. Dębska et al [25] showed that adding waste glass into epoxy mortars can improve its compressive and flexural strengths and absorbability. Formisano et al [26] showed that adding microscopic silicon carbide powders in different contents and aggregate sizes could improve the low abrasive wear resistance of an epoxy resin system. Laboratory testing results show that resins with some admixture incorporation possess improved compressive and shear strengths; and pullout testing results show that resin anchoring agent mixed admixture can increase the load bearing capacity of fully grouted rebar bolts [27–31].

**Fig 1:**
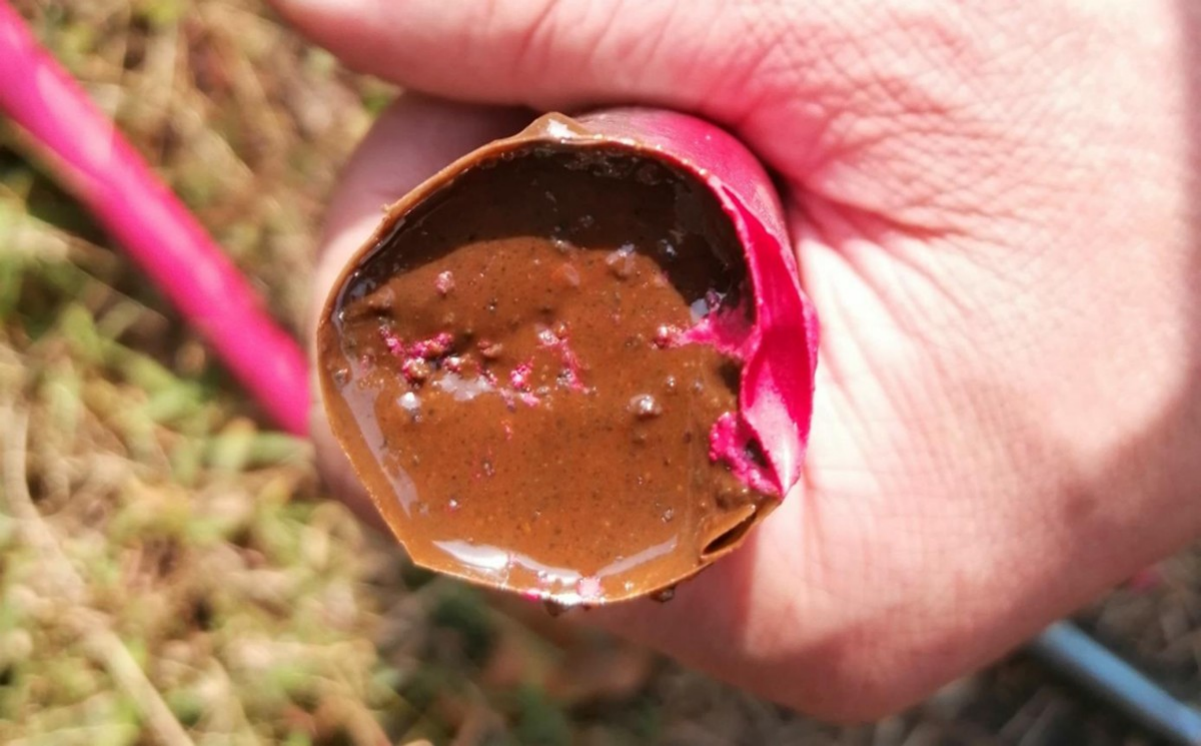
Cartridge resin anchoring agents using in coal mines.

The above research show that adding aggregates to grouting materials improves their strength and load bearing capacity. However, resin anchoring agents currently used in coal mines are mainly small aggregates such as cement or marble powder, without larger aggregates, as shown in Fig 1. Therefore, it is necessary to modify the grouting material to improve bolt support in coal mines.

## Theory and experimental methods

### Theory

**Fig 2:**
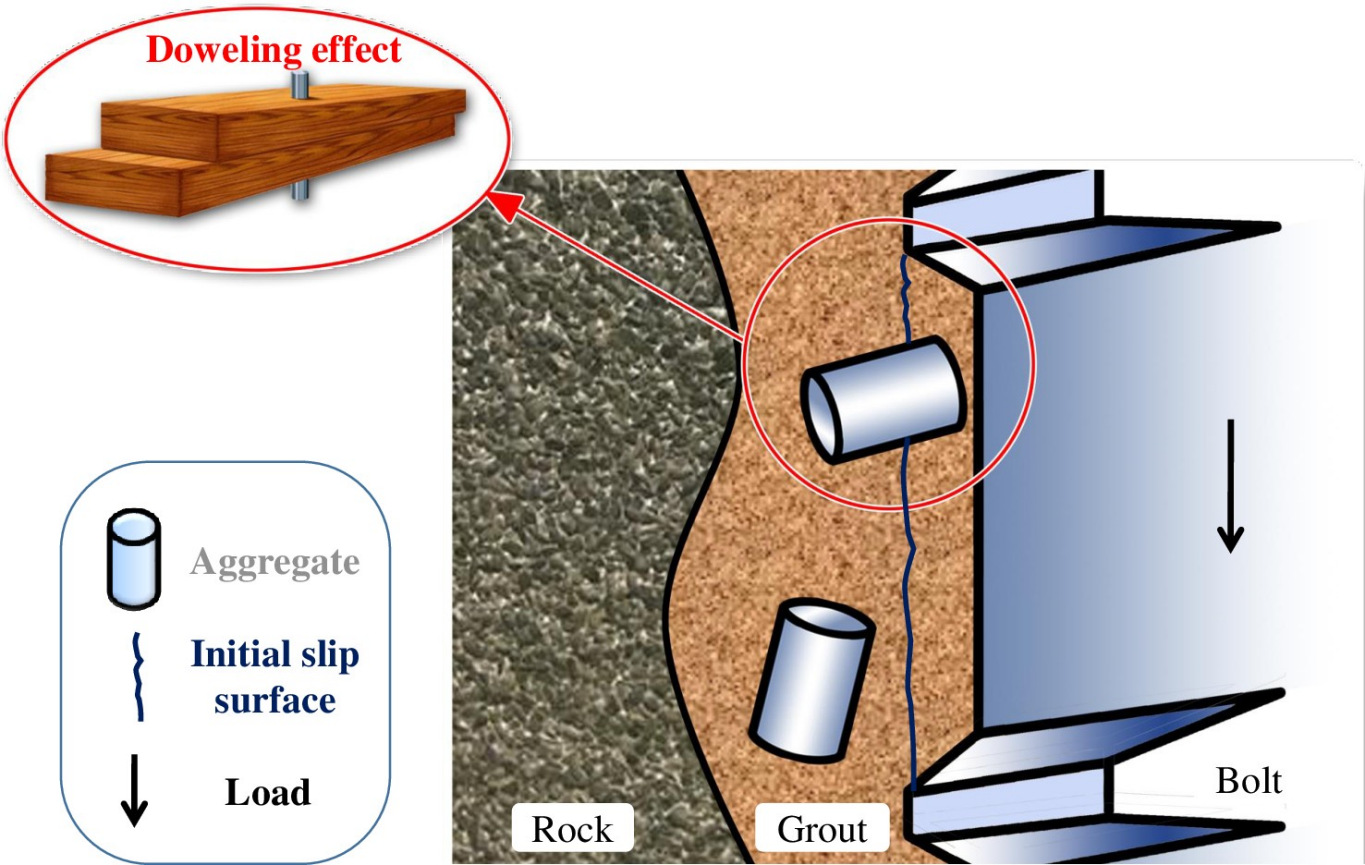
Working principle of mixing metallic aggregates into grout [36].

Experimental observations [32–34] have confirmed that resin shear failure along a cylindrical surface around the bolt/resin interface is a predominant failure mode in such reinforcement systems. These studies show that for a bolting system, the axial load capacity depends on the shear resistance generated at the resin-bolt interface, whereas the shear stress at the failure interface is the result of bonding, friction and mechanical interlocking. It can be deduced that the reinforcement effect of the whole anchoring system can be directly enhanced by improving the shear capacity of the resin anchoring agent at the failure interface. The increase in anchorage force as a result of adding aggregates into grouting materials can be explained as that whenever an aggregate presents at the failure surface, it acts as an interlock to resistance against expansion of the failure surface; thus, the aggregate produces a doweling effect [35]. If one or more rigid aggregates are present on the slip surface, as shown in Fig 2, a doweling effect can be created by them. Consequently, the slip surface can no longer be the former regular cylindrical surface and extra radial dilation has to be generated due to the interactions between the aggregate-grout matrix and aggregate-bolt ribs, thereby resulting in change of the failure mode. Therefore, the axial loading capacity of the bolt can be improved due to a higher confining pressure being generated at the slip surface.

In a previous experimental study [36,37], a steel wire with a diameter of 2.0 mm was cut manually into lengths of 2.0 to 3.5 mm and these were used as aggregates for the resin anchoring agent. Experimental results showed that the average incremental load of the bond was about 14%, and the average increment of energy absorption was about 70% of the specimens with steel aggregates. This suggests that the load transfer capacity of the anchor system can be improved using the proposed resin modification.

However, there is no quantitative conclusion on the optimum resin modification with respect to anchoring performance. Therefore, this study investigates the mechanical properties of resins with different aggregate shapes, sizes and quantities, and their effects on bolting performance.

### Methods

To investigate the optimum steel aggregate of resin anchoring agents, six kinds of steel aggregates were selected and tested. Uniaxial compressive and direct shear tests were conducted to evaluate the mechanical properties of resin materials with and without aggregates. Right spiral bolts, which were commonly used in underground coal mines in China, were used as testing bolts. Three tensile tests were also conducted to evaluate the axial deformational behaviour of the bolts.

The anchorage performance of resins with added steel aggregates were evaluated by pullout tests. A testing specimen is composed of one 280 mm length right spiral bolt, confined in a 100 mm long and 7.0 mm thick steel tube by slow gel time resin with or without steel aggregates. The main observations in the pullout test included ultimate pullout force, energy consumed by the axial force, and circumferential strain of the steel tube. The ultimate pullout force of the bolting specimen represents the axial load capacity of the anchorage. The energy consumption refers to the work done by the pullout force during testing, which is calculated using the enclosed area of the pullout curve. The circumferential strain of the steel tube was also measured during the testing procedure to investigate the radial dilation of the bolting specimen. The experiment and testing numbers are shown in Table 1.

**Table 1:** Experiment and testing numbers.

Experiment	Number
Uniaxial compressive test	57
Direct shear test	57
Bolt tensile test	3
Pullout tests	60

## Materials and equipment

### Experimental materials

#### (1) Bolt

**Fig 3:**
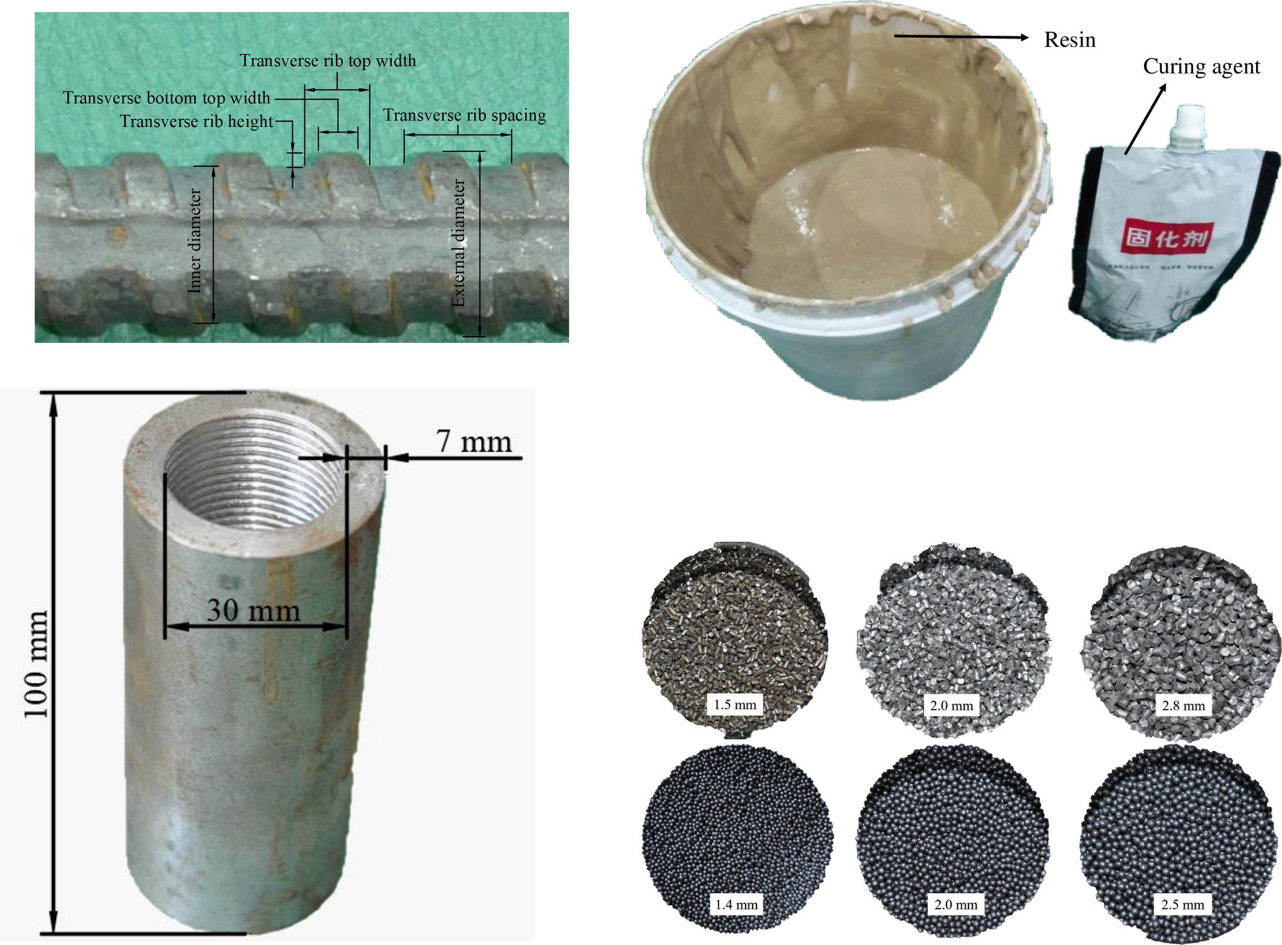
Experimental materials.

In this study, 20 mm diameter right spiral rebar bolts that are commonly used in underground coal mines in China are selected, as shown in Fig 3(a). The bolt profile configuration is shown in Table 2.

**Table 2:** Profile configurations of right spiral bolts.

Parameter	Value	Parameter	Value
Diameter/mm	20	Rib spacing/mm	12
Rib height/mm	1.8	Rib rising angle/(°)	78
Rib lateral angle/(°)	72	Rib width top/bottom /mm	4.3/5.7

#### (2) Steel tube

Results of previous studies [36,37] suggest that the impact of wall thickness of the confining tube is not significant; hence, only 7 mm thick tubes are selected in this study, as shown in Fig 3(B). The confining tube of the bolting specimen is made of a 100 mm long steel pipe with a wall thickness of originally 7.5 mm and an inner diameter of 30 mm, which refers to a borehole diameter drilled by a 28 mm drilling bit. The inner wall of the tube was threaded to prevent relative slip at the tube-resin interface. The equivalent wall thickness of the tube is approximately 7.0 mm due to 1.0 mm thread height.

#### (3) Resin

In the underground coal mining industry, encapsulated resin is used as grouting material. However, for easy casting and preparation of the bolting specimen, bulking packaged slow gel time resin, as shown in Fig 3(C), was tested and used as the grouting agent in this study. Its gel time is nominated as 20 min at a resin matrix to catalyst ratio of 1:0.04.

#### (4) Steel aggregates

Two kinds of steel aggregate shapes were used as resin aggregates, namely columnar steel grit and spherical steel shot, as shown in Fig 3(D). All steel grits are 2.0–3.0 mm in length, but possess different diameters of 1.5, 2.0 and 2.8 mm. The steel shots used in the tests have different aggregate diameters of 1.4, 2.0 and 2.5 mm. For each kind of steel aggregate, quantities of 30, 40 and 50 were tested to evaluate the effect of aggregate quantity. Three specimens were prepared for each test.

### Specimen preparation

#### (1) Uniaxial compressive strength testing specimen

**Fig 4:**
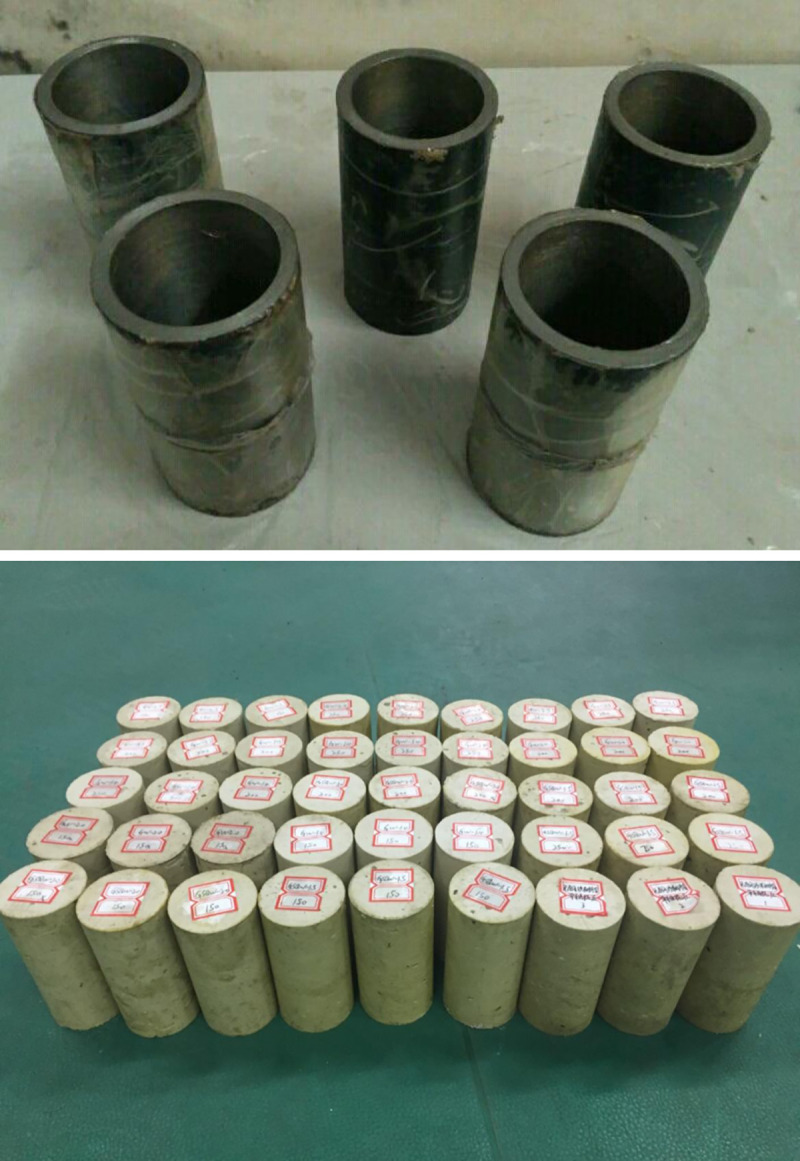
Compressive strength testing specimens.

According to the industrial standard MT146.1–2011 [38], resin specimens in the uniaxial compressive test were prepared using steel molds with dimensions of 50 mm diameter and 100 mm length, as shown in Fig 4(A). For specimens with steel aggregates, according to the aggregate quantities of 30, 40 and 50 per specimen, it can be calculated that the number of aggregates added to one compressive testing specimen is 150, 200 and 250, respectively. In the preparation of the testing specimens, lubricating oil was first applied onto the inner wall of the mold to facilitate later de-molding of the specimens, and the anchorage agent mixed with proper steel aggregates was stirred thoroughly and poured into the mold carefully. The specimens are shown in Fig 4(B).

#### (2) Direct shear testing specimen

**Fig 5:**
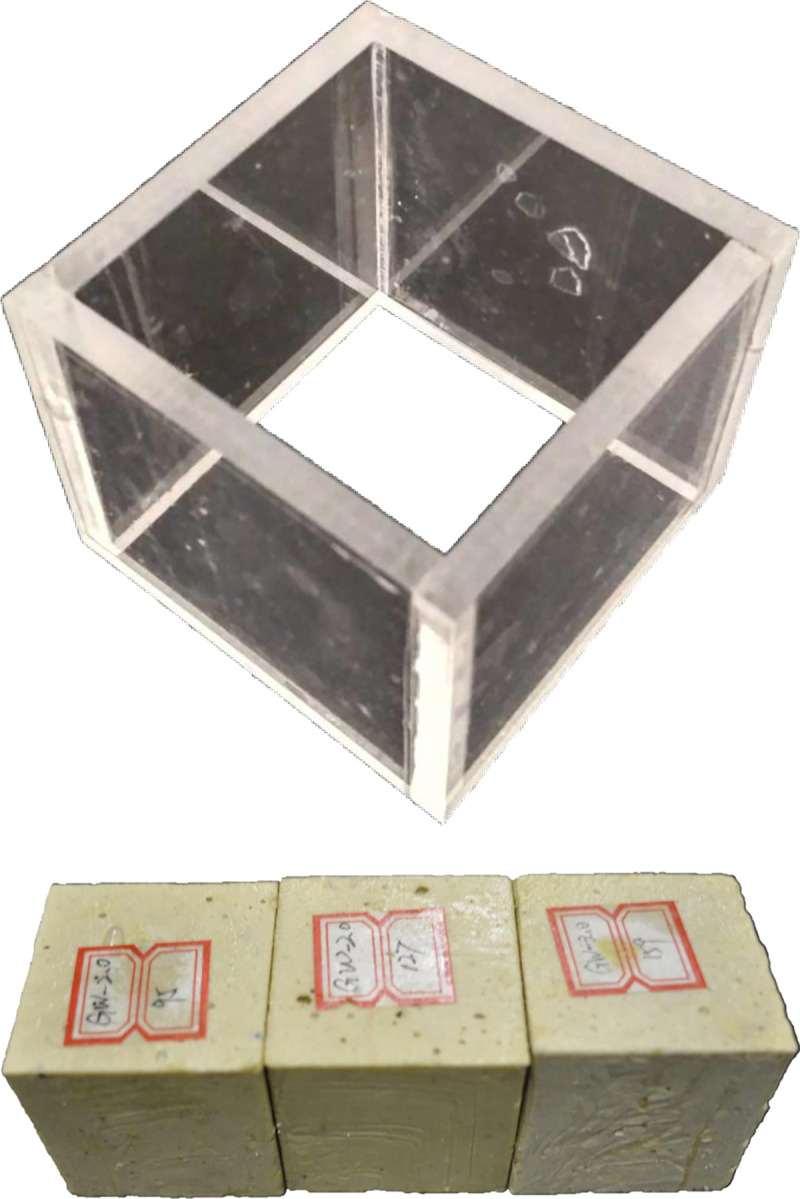
Compressive and shear specimens.

The direct shear testing specimens were prepared as 50 mm cubes, and the corresponding mold is shown in Fig 5(A). Specimens with 100, 130 or 160 added steel aggregates were equivalent to 30, 40 or 50 aggregates in one pullout specimen, respectively. Similar to making compressive specimens, the direct shear testing specimens are shown in Fig 5(B).

#### (3) Pullout specimen preparation

**Fig 6:**
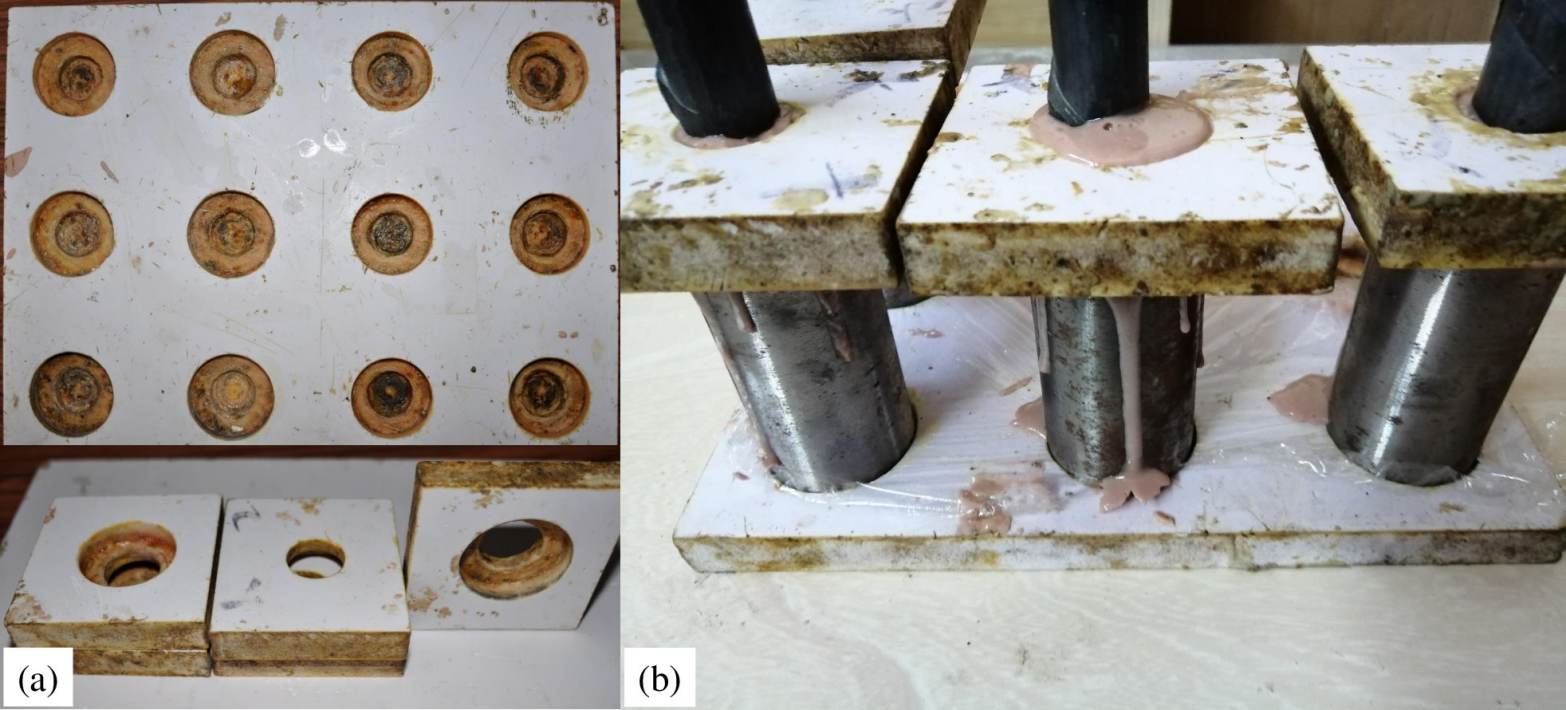
Centering device and bolting specimen.

To ensure that the bolt is centrally anchored in the steel tube, a set of devices, as shown in Fig 6(A), is designed to prepare the bolting specimen. Bulk-packed slow gel time mix and pour resin, with or without steel aggregates, were used as grouting material to encapsulate the bolt within the steel tubes, as shown in Fig 6(B).

**Fig 7:**
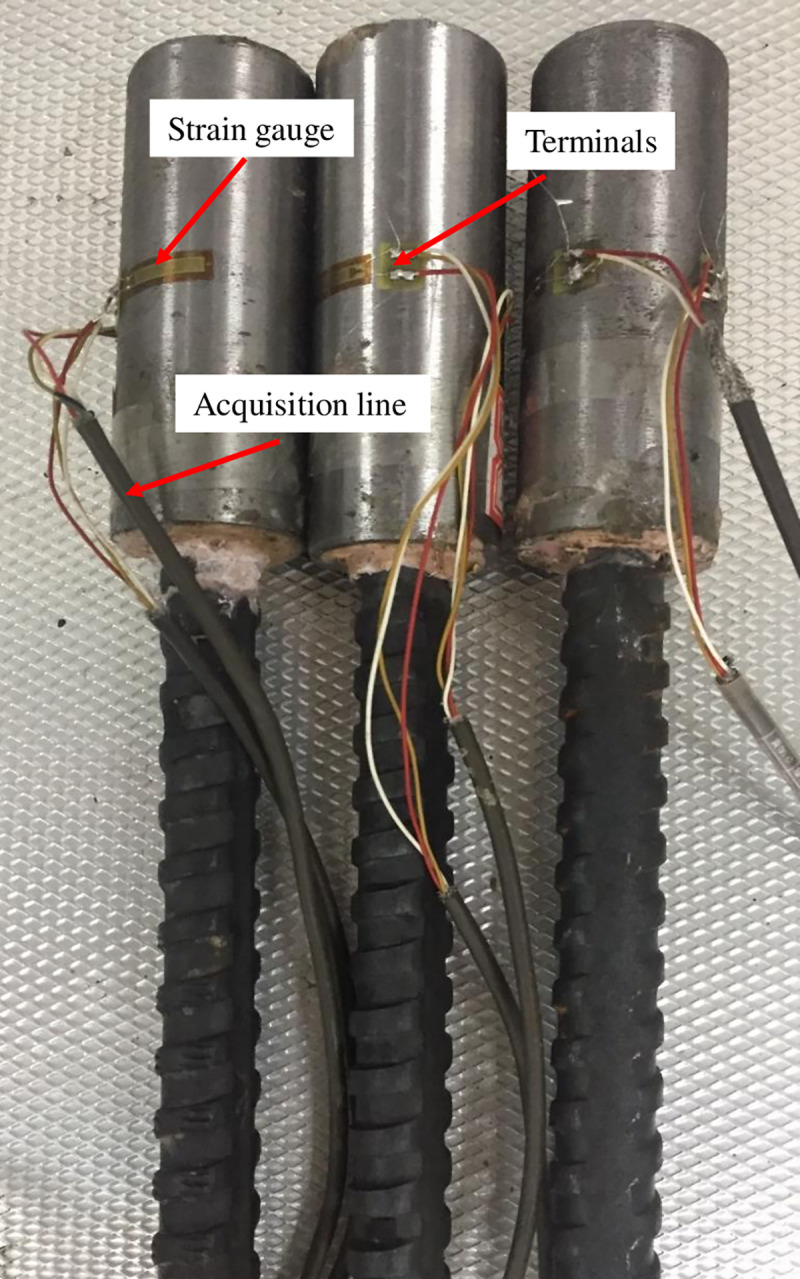
Anchorage specimen.

The bolting specimens were placed in an incubator of 22°C for 2 h after the resin was consolidated. Then, two strain gauges were opposite stuck onto the middle of the steel tube for circumferential strain measurement, as shown in Fig 7.

### Testing equipment

#### (1) Holding device

**Fig 8:**
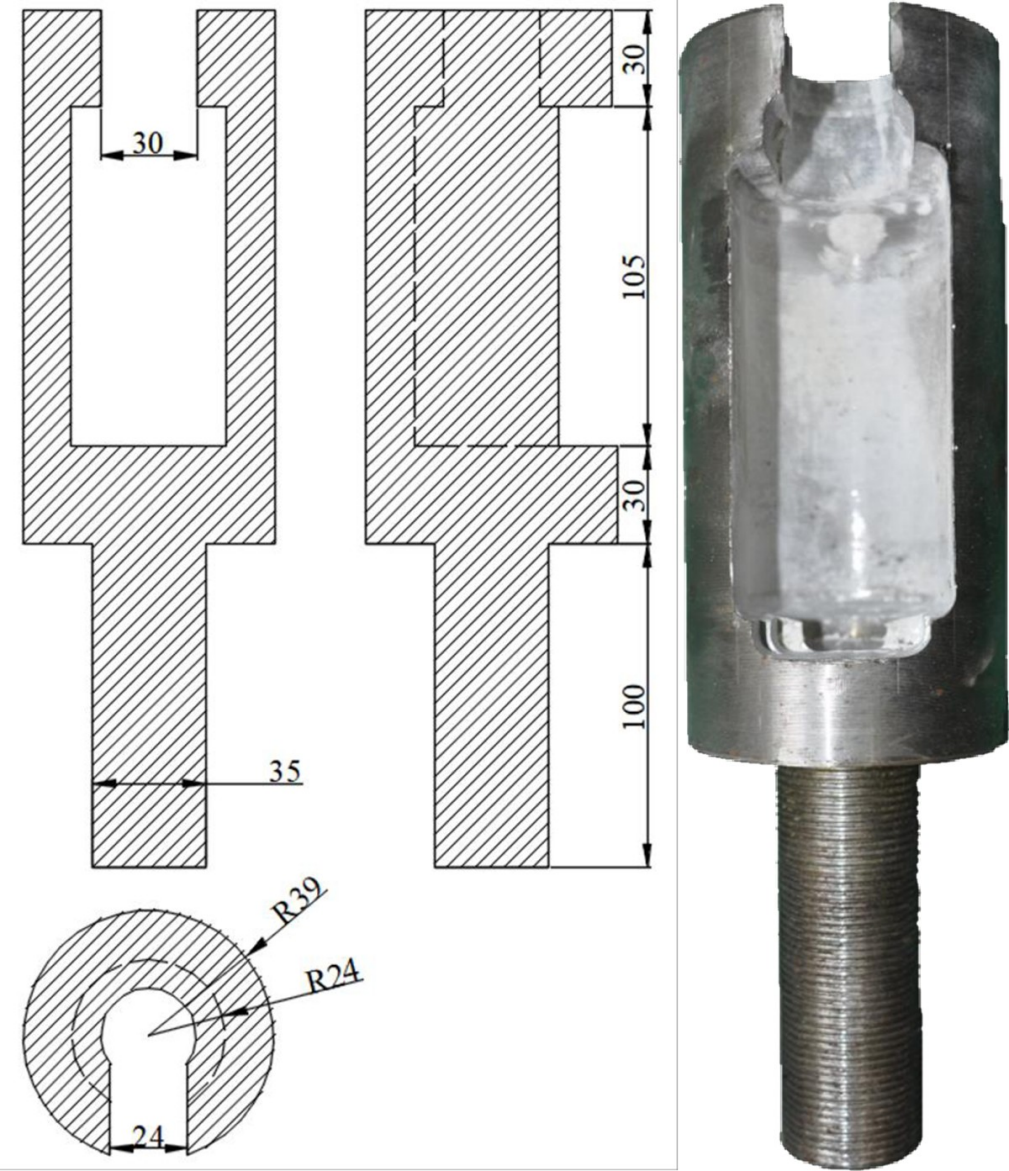
Pullout specimen holding device (unit: mm).

A bolting specimen holding device has been designed and milled using 40 cr alloy steel with a yield strength of 785 MPa. The dimensions of the device are shown in Fig 8.

#### (2) Testing machine

**Fig 9:**
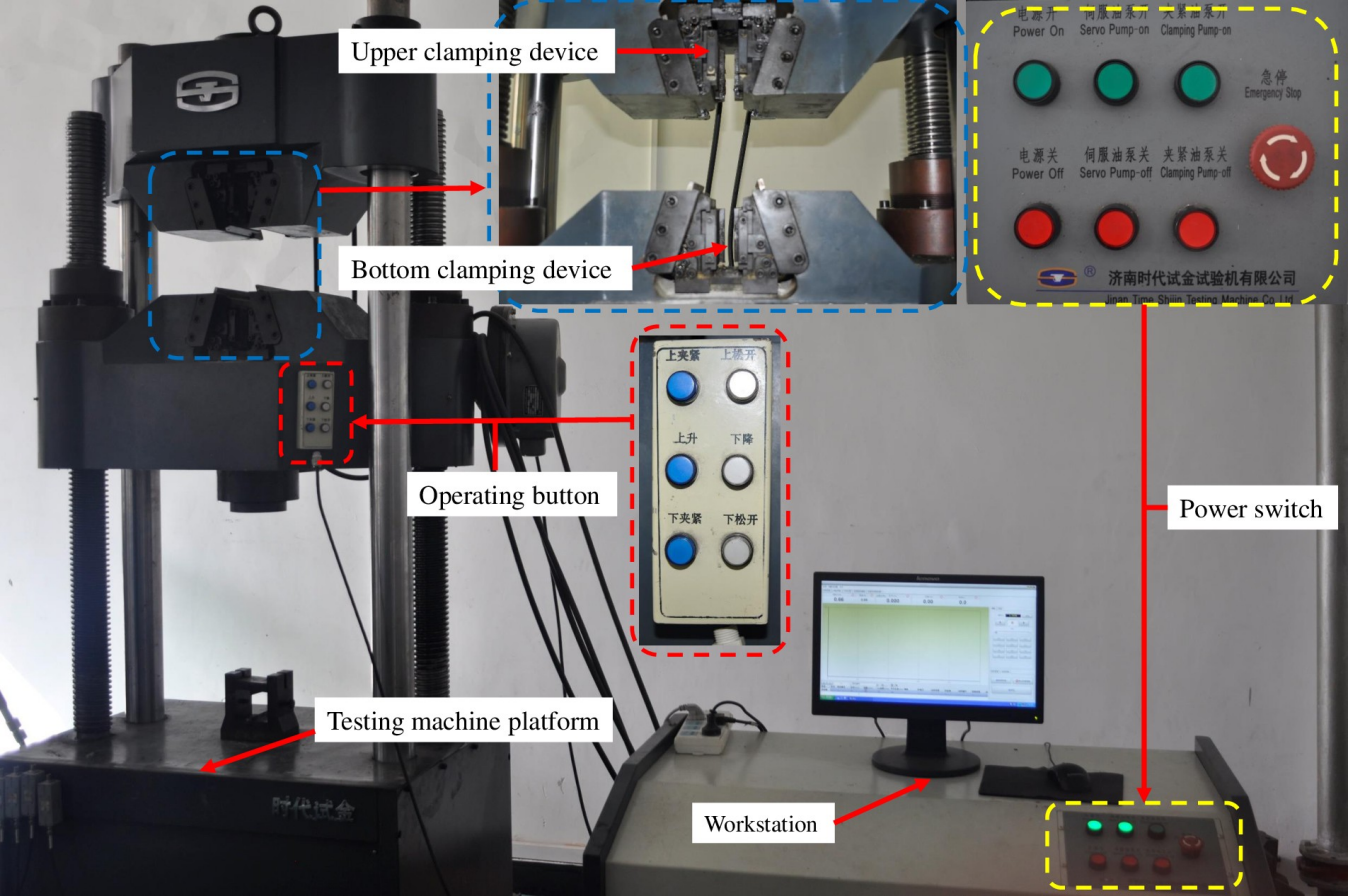
WAW-600C electro-hydraulic servo universal testing machine.

Microcomputer controlled WAW-600C electro-hydraulic servo universal testing machine was used for the bolt tensile tests, uniaxial compressive tests and direct shear tests of the resin, and pullout tests of the bolting specimens, as shown in Fig 9. Displacement control was used to realize the load in the tests; the loading rate was set as 1 mm/min.

#### (3) DH5929 data logger

**Fig 10:**
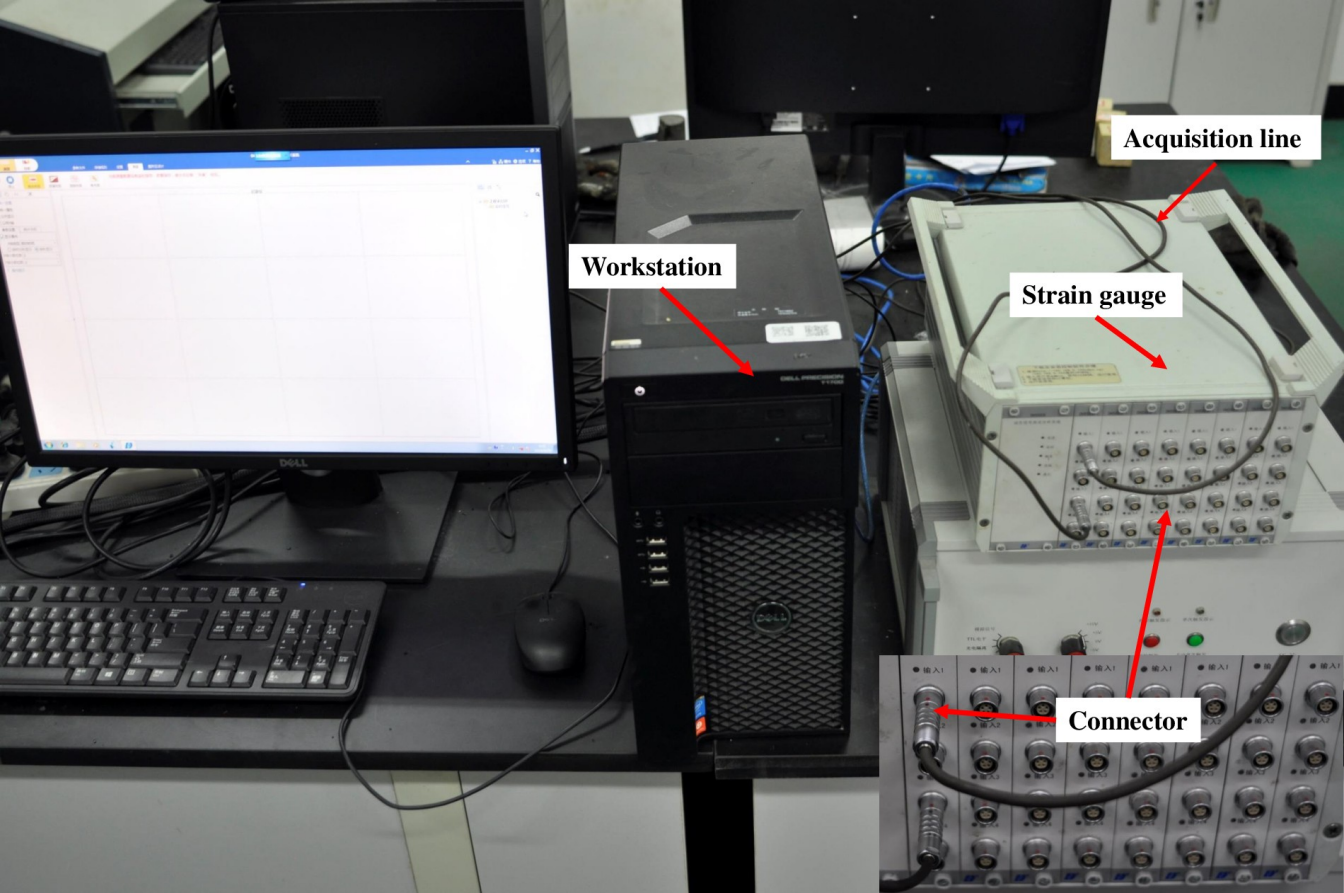
DH5929 strain logger.

DH5929 dynamic data logger was used to collect the circumferential strain data of the steel tube, as shown in Fig 10. In pullout tests, the data sampling frequency was 2 Hz.

## Results

### Bolt tensile tests

**Fig 11:**
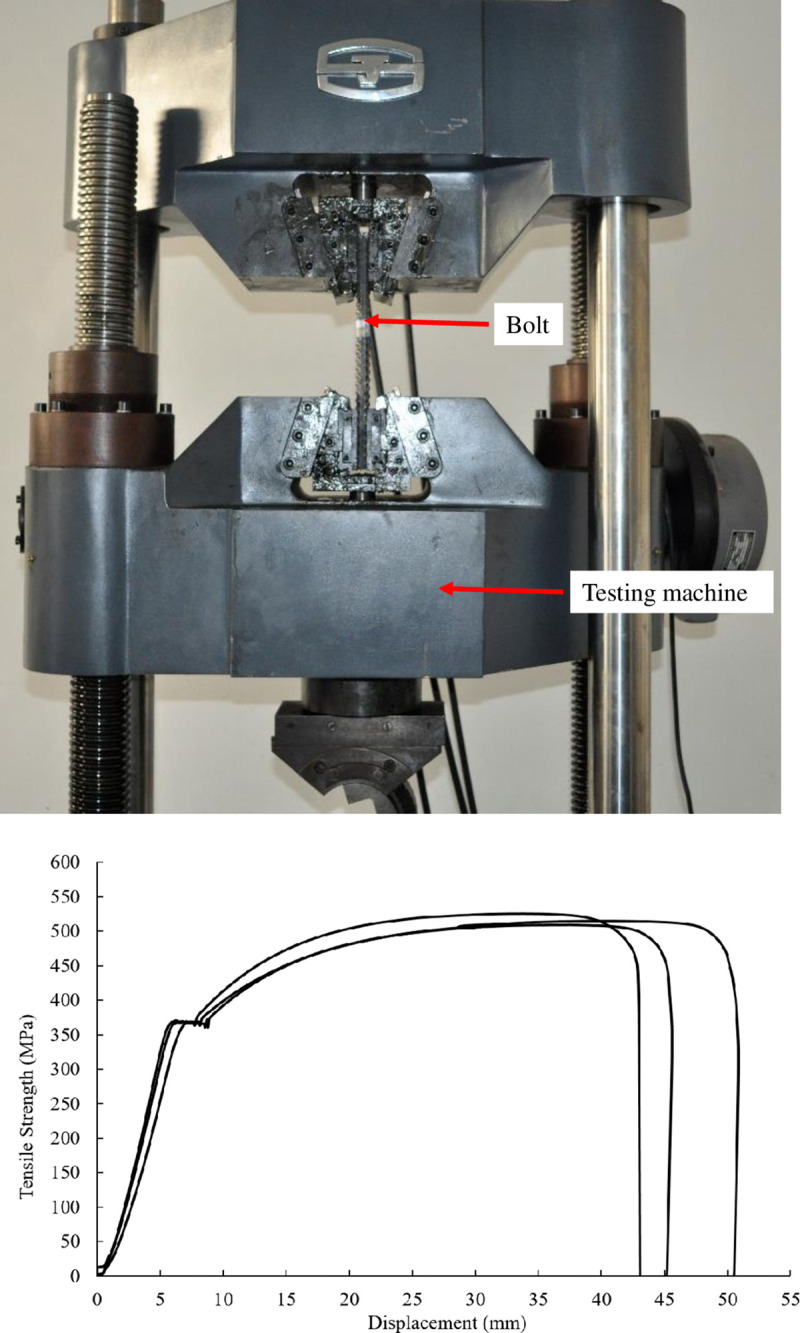
Tensile test and results (a) Tensile test. (b) Tensile curve of the bolt.

The axial deformational behaviour of the steel bolt was measured, as shown in Fig 11(A). Three bolts were tested, and it was found that the average yielding strength of the bolt was 403.1 MPa (126.6 kN), and the average peak strength was 567.5 MPa (178.3 kN), as shown in Fig 11(B).

### Axial compressive tests

**Fig 12:**
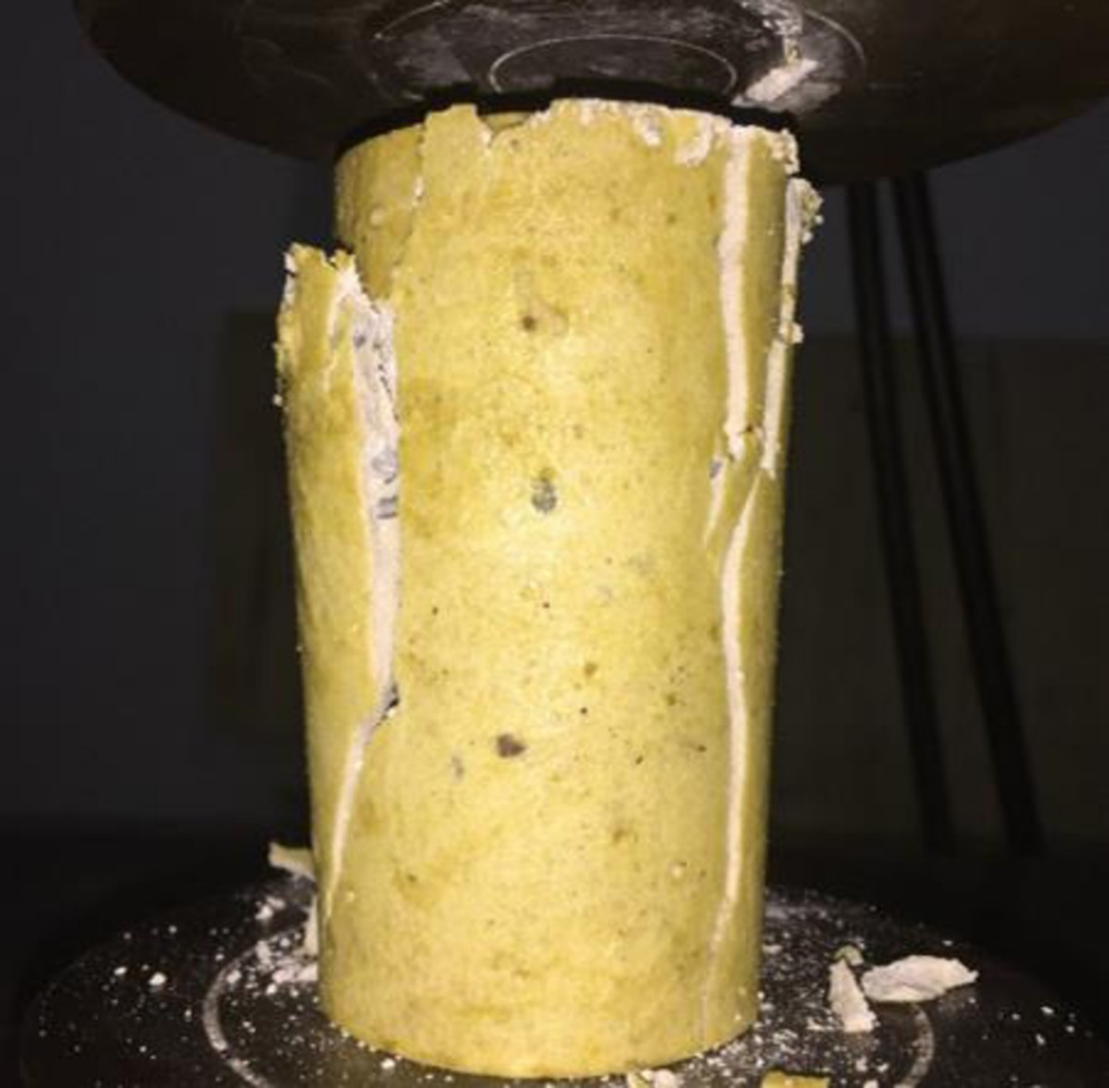
Uniaxial compressive test.

The axial compressive strength test of the resin, with or without steel aggregates, were conducted using WAW-600C, as shown in Fig 12. It was found that the average uniaxial compressive strength of the resin specimens without aggregates was 58.3 MPa; and the average strength of resin specimens with steel grits and steel shots was 63.2 and 65.2 MPa, respectively, which represented 8.4% and 11.8% strength increases compared to pure resin specimens.

### Direct shear tests

**Fig 13:**
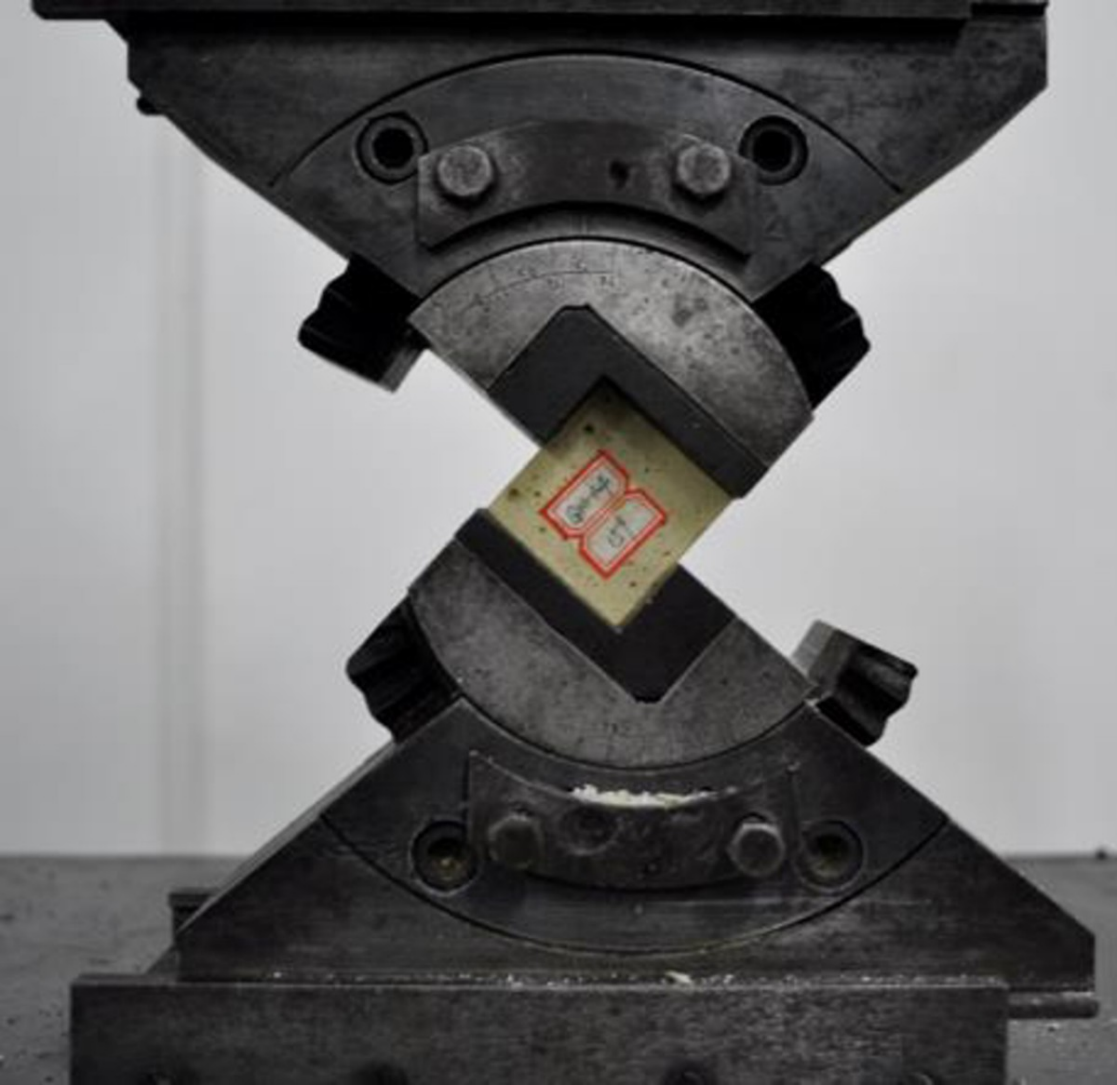
Direct shear test.

Direct shear tests were carried out, as shown in Fig 13. The average shear strength of the pure resin anchoring agent was measured as 27.7 MPa; and the average shear strengths of resin specimens with steel grits and steel shots were 32.4 and 31.4 MPa, respectively, which represented 17.0% and 13.4% increases compared to the pure resin specimen.

### Pullout tests

**Fig 14:**
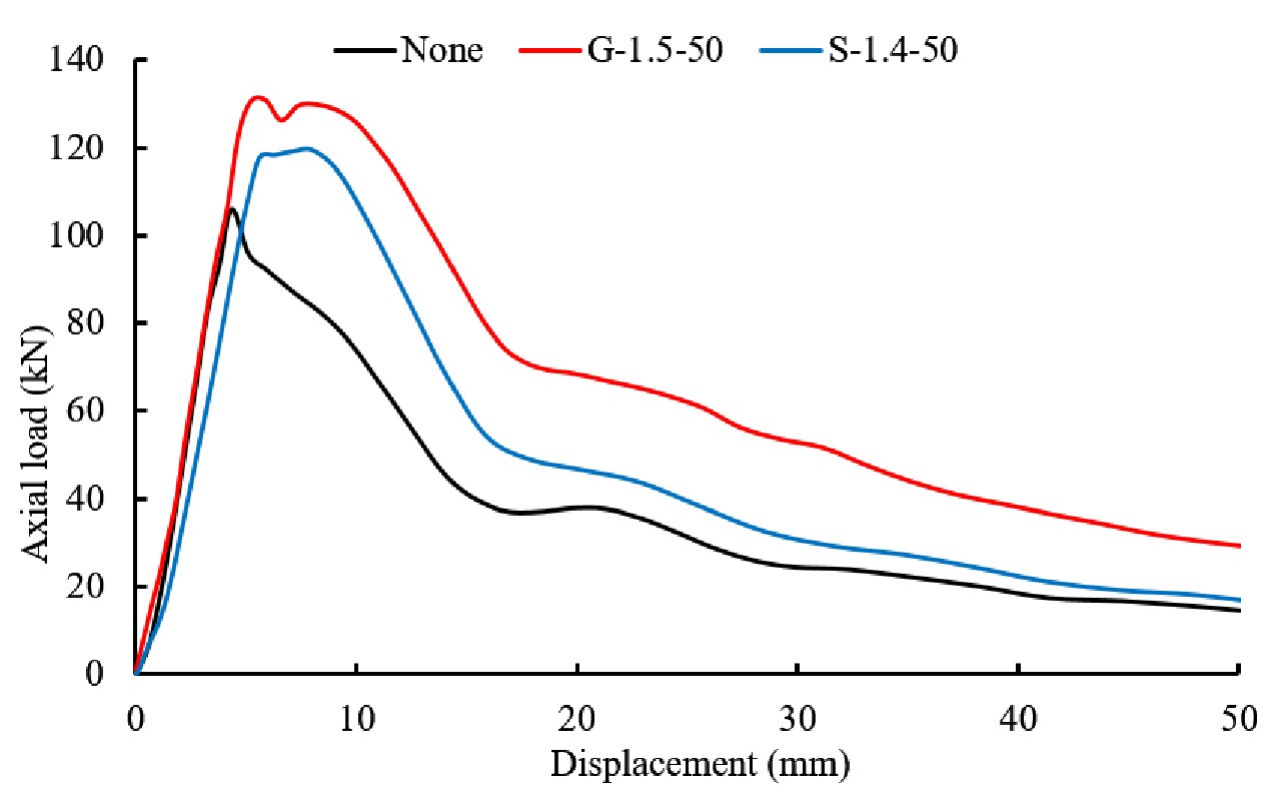
Typical load-displacement curves of the test (G = Grit, S = Shot).

Pullout tests were conducted to evaluate the anchorage performance of bolting specimens with and without steel aggregates. Typical load-displacement curves in the test are shown in Fig 14 where ‘None’ represents bolting specimens without aggregates, G-1.5–50 represents specimens with 50 1.5 mm diameter grits, and S-1.4–50 refers to specimens with 50 1.4 mm diameter shots.

The average peak load of each kind of bolting specimen was calculated and summarized in Table 3. The circumferential strain of the steel tube was measured and the energy consumption of the pullout force was calculated by the area enclosed by the pullout curve. In addition, the percentage increments with respect to the bolting specimen without aggregates were calculated and presented. All pullout curves are attached in S1 and S2 Figs.

**Table 3:** Testing results of the pullout specimens.

Aggregates	Size /mm	Quantity	Average peak load /kN	Increments %	Average cir. strain /με	Increments %	Average energy consumption /J	Increments %
None	--	--	119.2	0	282.8	0	2420.5	0
Grit	1.5	30	123.8	3.9	371.3	31.3	2617.0	8.1
		40	131.3	10.2	324.5	14.7	2746.8	13.5
		50	126.8	6.4	317.5	12.3	2991.7	23.6
	2.0	30	124.0	4.0	350.0	23.8	2443.7	1.0
		40	123.4	3.5	253.7	-10.3	2703.7	11.7
		50	122.7	2.9	415.7	47.0	2324.8	-4.0
	2.8	30	129.9	9.0	417.0	47.5	3071.5	26.9
		40	130.1	9.1	538.8	90.5	3322.5	37.3
		50	123.8	3.9	457.8	61.9	2664.5	10.1
Shot	1.4	30	135.1	13.3	292.8	3.5	2827.0	16.8
		40	132.2	10.9	266.3	-5.8	2432.3	0.5
		50	126.8	6.4	427.8	51.3	3237.7	33.8
	2.0	30	124.8	4.7	372.0	31.5	3098.3	28.0
		40	123.5	3.6	338.5	19.7	2477.3	2.3
		50	129.9	9.0	361.2	27.7	2690.3	11.1
	2.5	30	133.8	12.2	375.3	32.7	3260.0	34.7
		40	128.0	7.4	401.7	42.0	3017.0	24.6
		50	132.0	10.7	386.8	36.8	3312.7	36.9

## Analysis and discussion

### Effect of aggregate quantity on anchorage performance

**Fig 15:**
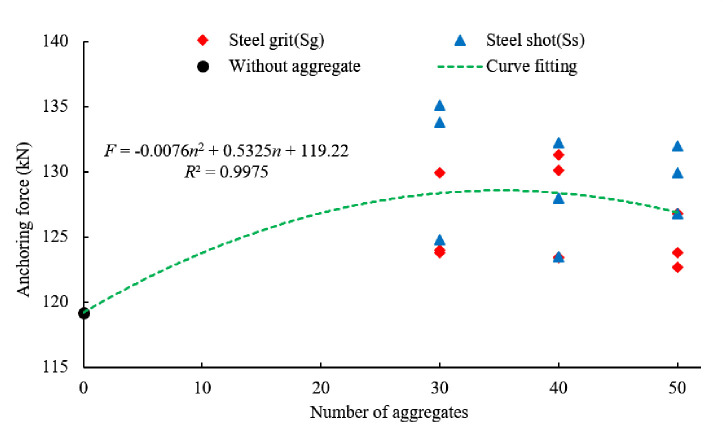
Scatter plot of average anchoring force and fitting curve of different numbers of aggregates.

Fig 15 shows the average ultimate anchoring force of steel grit aggregate numbers of 30, 40, and 50. By fitting the curve, the equation between the anchoring force and the quantity of aggregates in the experiment can be expressed as:

$$F=‐0.0076n^{2}+0.5325n+119.2$$ (1)

Where *F* is the anchoring force (kN) and *n* is the quantity of the aggregates.

Ignoring the shape and size of the steel aggregates, the average anchoring force of the specimens with 30, 40 and 50 steel aggregates increases by 7.9%, 7.5% and 6.5%, respectively.

**Fig 16:**
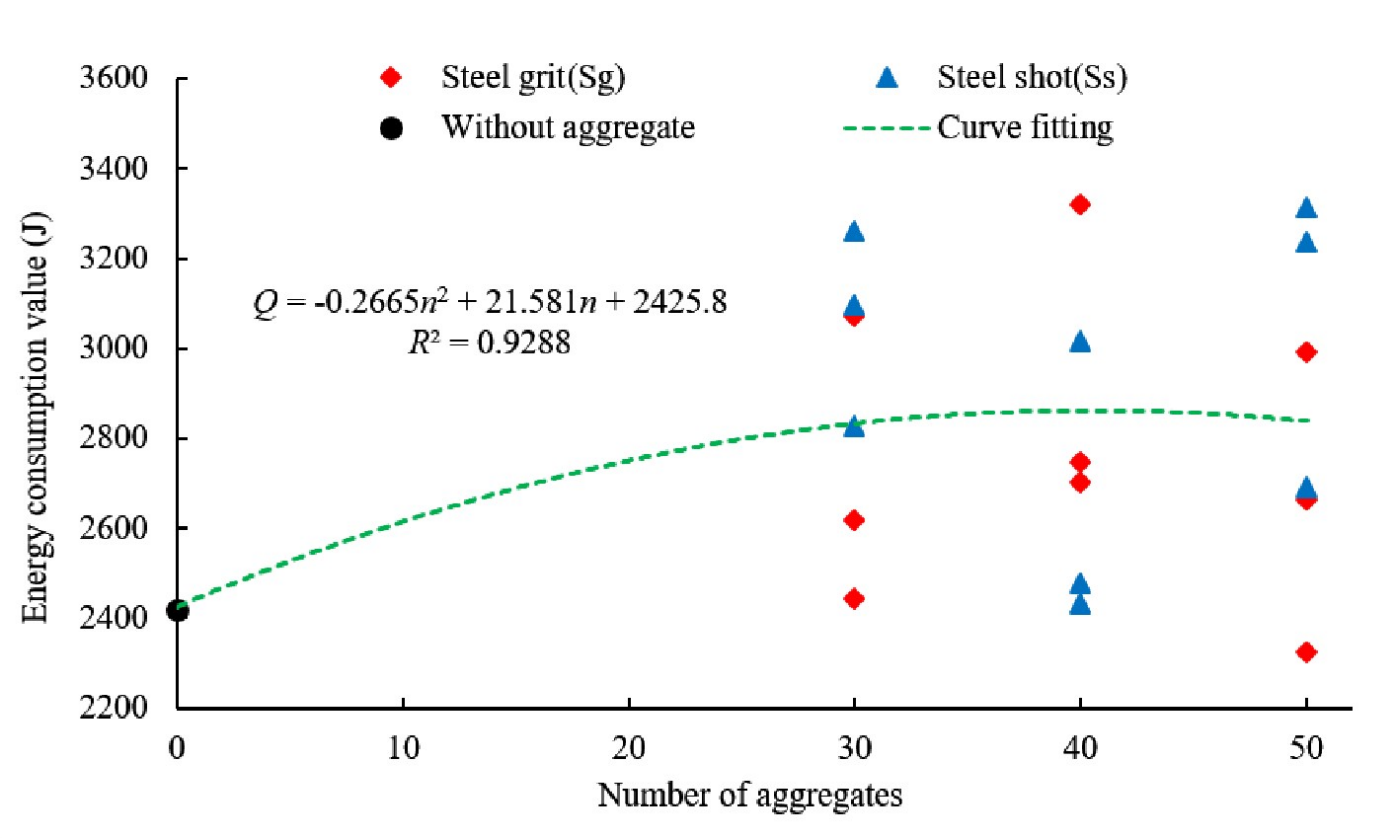
Energy consumption and fitting curve for aggregate quantity.

Fig 16 shows the average energy consumption for the steel grit quantities of 30, 40, and 50, respectively. By fitting the curve, the equation between the energy consumption and the aggregate quantity in the experiment is:

$$Q=‐0.2665n^{2}+21.58n+2426$$ (2)

Where *Q* is the energy consumption (J).

Ignoring the shape and size of the steel aggregates, the average energy consumption of the specimens with 30, 40 and 50 aggregates increases by 19.2%, 15.0%, and 18.6%, respectively.

**Fig 17:**
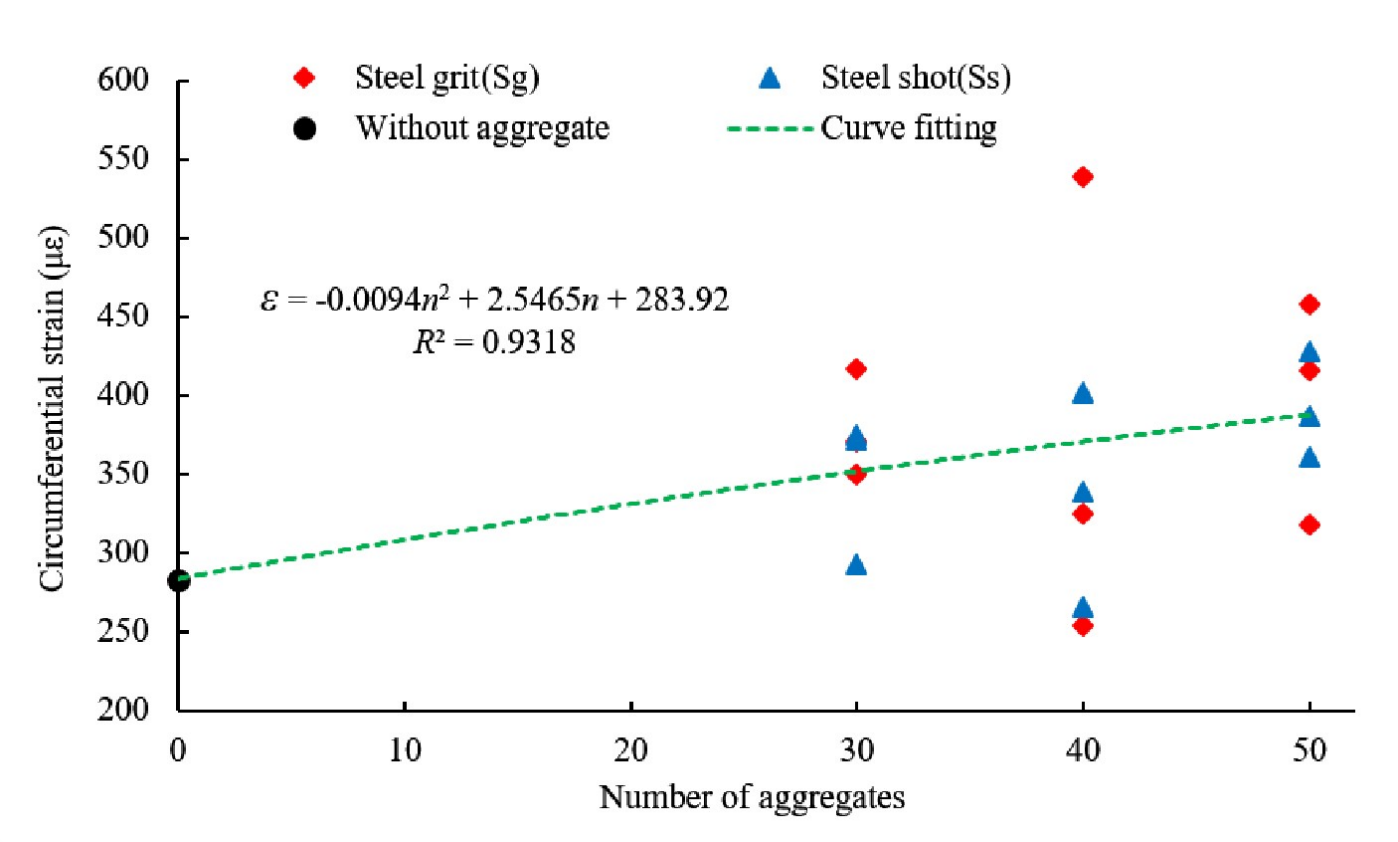
Scatter plot of circumferential strain and fitting curve of different aggregate quantities.

Fig 17 shows the circumferential strain of the sleeve with steel grit quantities of 30, 40, and 50, respectively. By curve fitting, the circumferential strain is associated with the aggregate quantity in the experiment by:

$$\varepsilon =‐0.0094n^{2}+2.547n+283.9$$ (3)

Where *ε* is the circumferential strain (με).

Ignoring the shape and size of the steel aggregates, the average circumferential strain of the specimens with 30, 40 and 50 aggregates increases by 28.4%, 25.1% and 39.5%, respectively.

### Effect of aggregate size on anchorage performance

**Fig 18:**
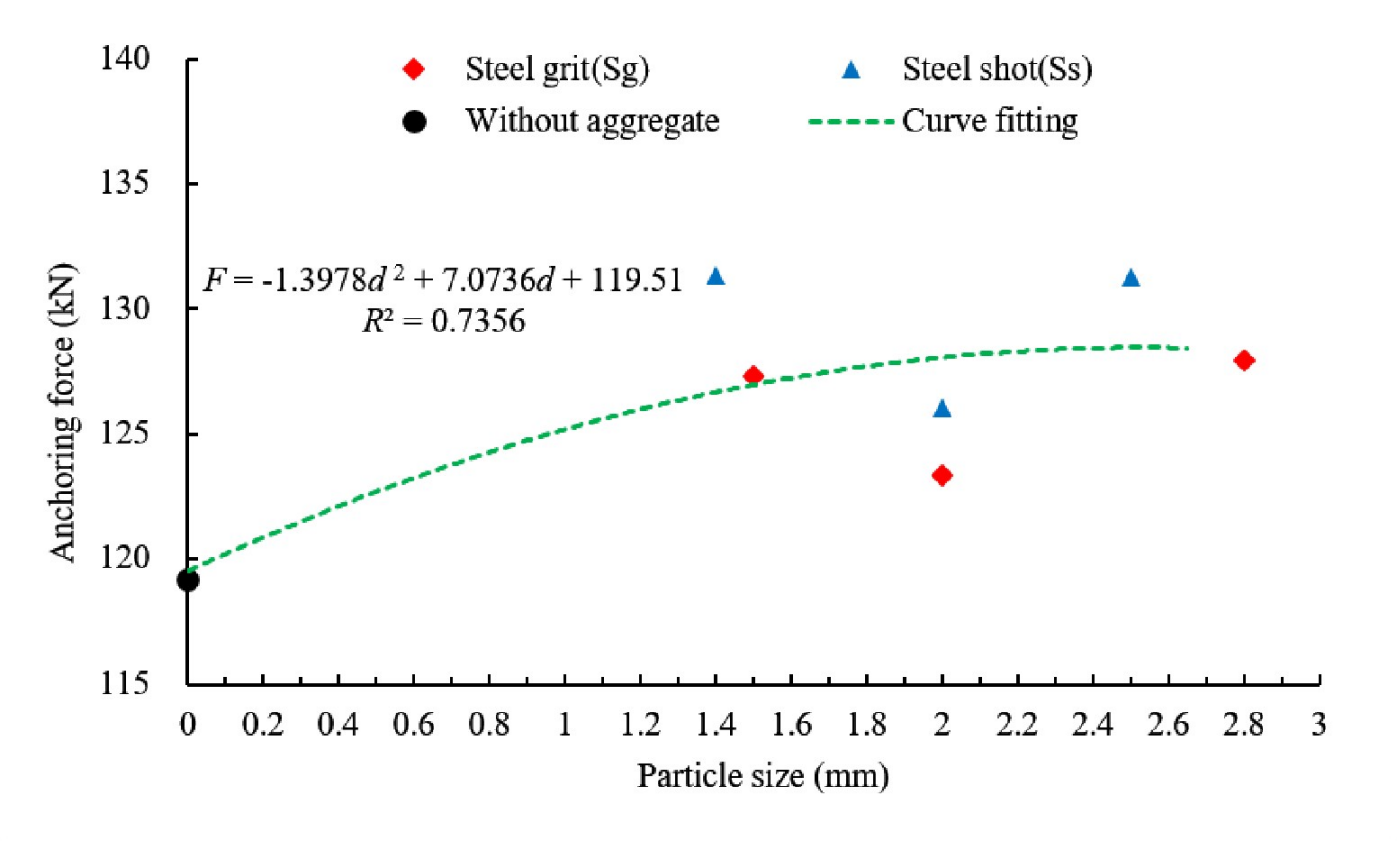
Scatter plot of average anchoring force and fitting curve of different aggregate sizes.

Fig 18 show the relationship between average anchoring force and size of the steel aggregates. By curve fitting, the relationship between the anchoring force and the aggregate size (diameter) in the experiment is:

$$F=-1.398d^{2}+7.074d+119.5$$ (4)

Where *d* is the size (diameter) of aggregates.

Ignoring the shape and quantity of the steel aggregates, the average anchoring force of the specimens with aggregate sizes (diameters) of 1.4~1.5, 2.0~2.0, and 2.5~2.8 mm increases by 8.5%, 4.6% and 8.7%, respectively.

**Fig 19:**
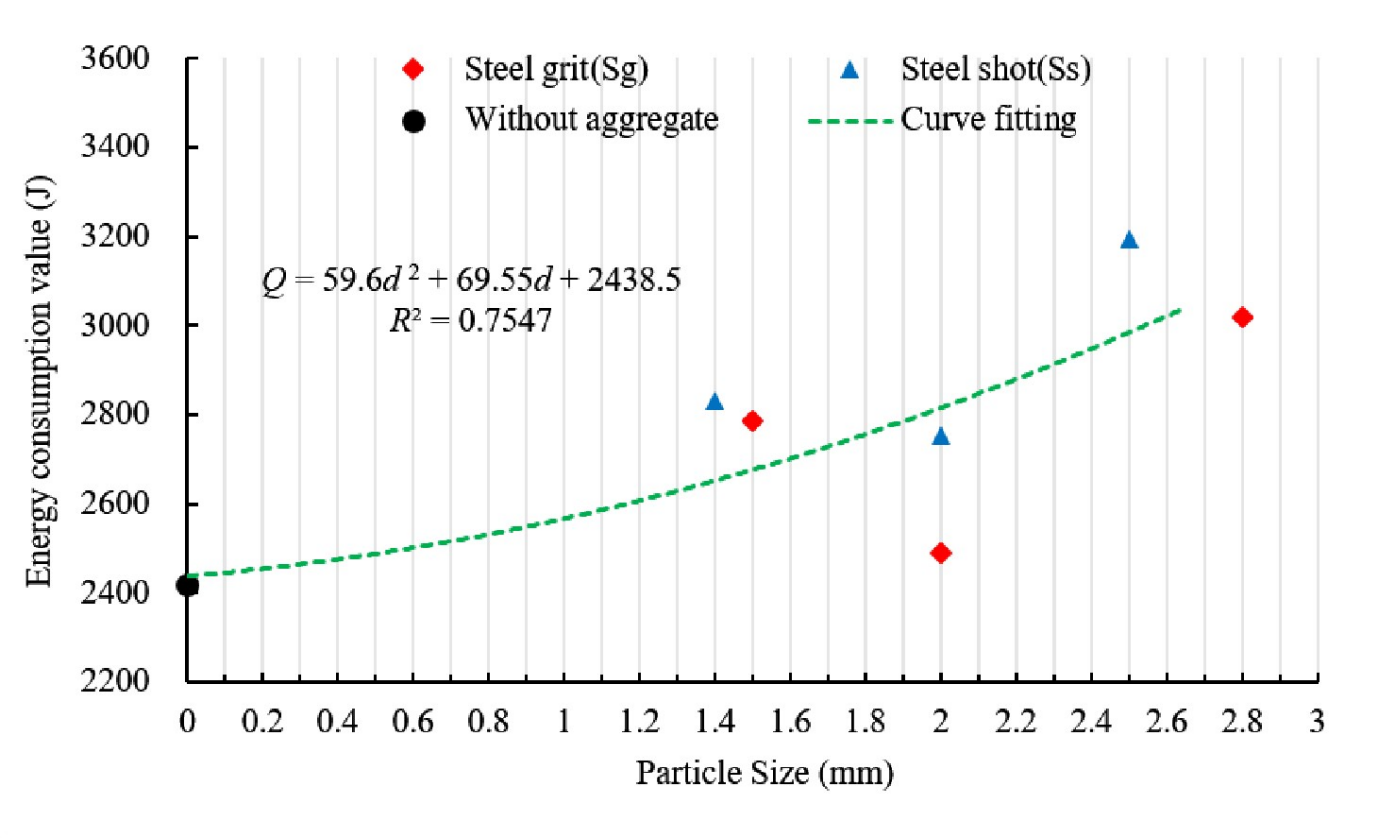
Scatter plot of energy consumption and fitting curve of different aggregate sizes.

Fig 19 shows the relationship between energy consumption and aggregate size. By curve fitting, the equation between the energy consumption and the aggregate size (diameter) in the experiment can be expressed as:

$$Q=59.6d^{2}+69.55d+2439$$ (5)

Ignoring the shape and quantity of the steel aggregates, the average energy consumption of the specimens with aggregate sizes (diameters) of 1.4~1.5, 2.0~2.0, and 2.5~2.8 mm increases by 16.0%, 8.4%, and 28.4%, respectively.

**Fig 20:**
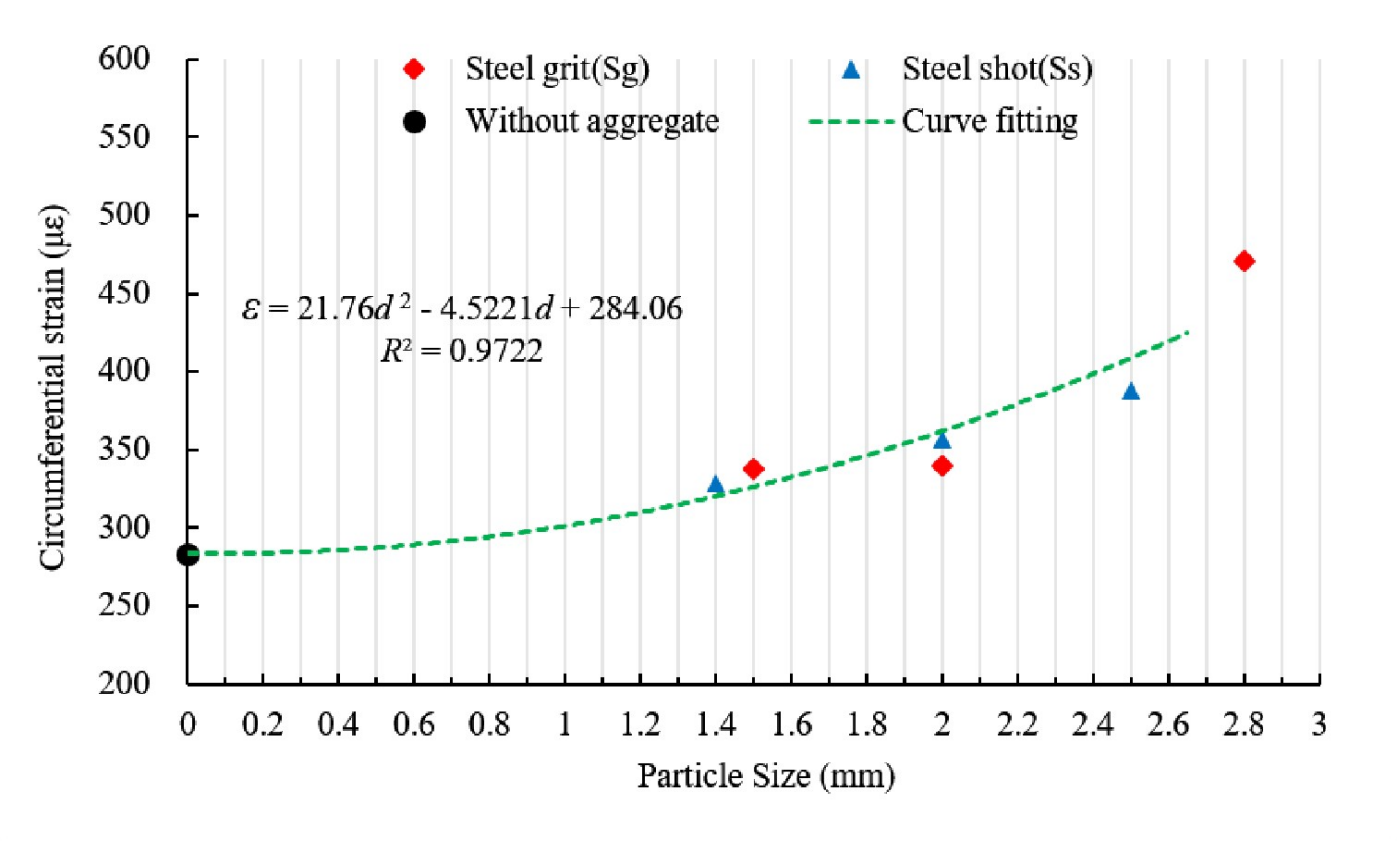
Scatter plot of circumferential strain and fitting curve of different aggregate sizes.

Fig 20 shows the relationship between the circumferential strain of the sleeves and aggregate size. By curve fitting, the circumferential strain and the aggregate size (diameter) are linked by:

$$\varepsilon =21.76d^{2}-4.5221d+284.06$$ (6)

Ignoring the shape and quantity of the steel aggregates, the average circumferential strain of the specimens with aggregate sizes (diameters) of 1.4~1.5, 2.0~2.0, and 2.5~2.8 mm increases by 17.9%, 23.3% and 51.9%, respectively.

As shown in Table 4, the fitting formulae can describe the relationships of anchoring force, energy consumption, and circumferential strain with aggregate quantity and size. This shows that the developed equations can be used to determine the performance of a specific bolt with different aggregate quantities and sizes, and provide accurate parameters for numerical modelling.

**Table 4:** Relationships of the anchoring force, energy consumption, and circumferential strain with aggregate quantity and size.

Relationship	Fitting Equation	*R*^*2*^
Anchoring Force with Aggregate Quantity	*F* = -0.0076*n*^2^+0.5325*n*+119.2	0.9975
Energy Consumption with Aggregate Quantity	*Q* = -0.2665*n*^2^+21.58*n*+2426	0.9288
Circumferential Strain with Aggregate Quantity	*ε* = -0.0094*n*^2^+2.547*n*+283.9	0.9318
Anchoring Force with Aggregate Size	*F* =−1.398*d*^2^+7.074*d*+119.5	0.7356
Energy Consumption with Aggregate Size	*Q* = 59.6*d*^2^+69.55*d*+2439	0.7547
Circumferential Strain with Aggregate Size	*ε* = 21.76*d*^2^−4.5221*d*+284.06	0.9722

### Effect of aggregate shape on anchorage performance

**Fig 21:**
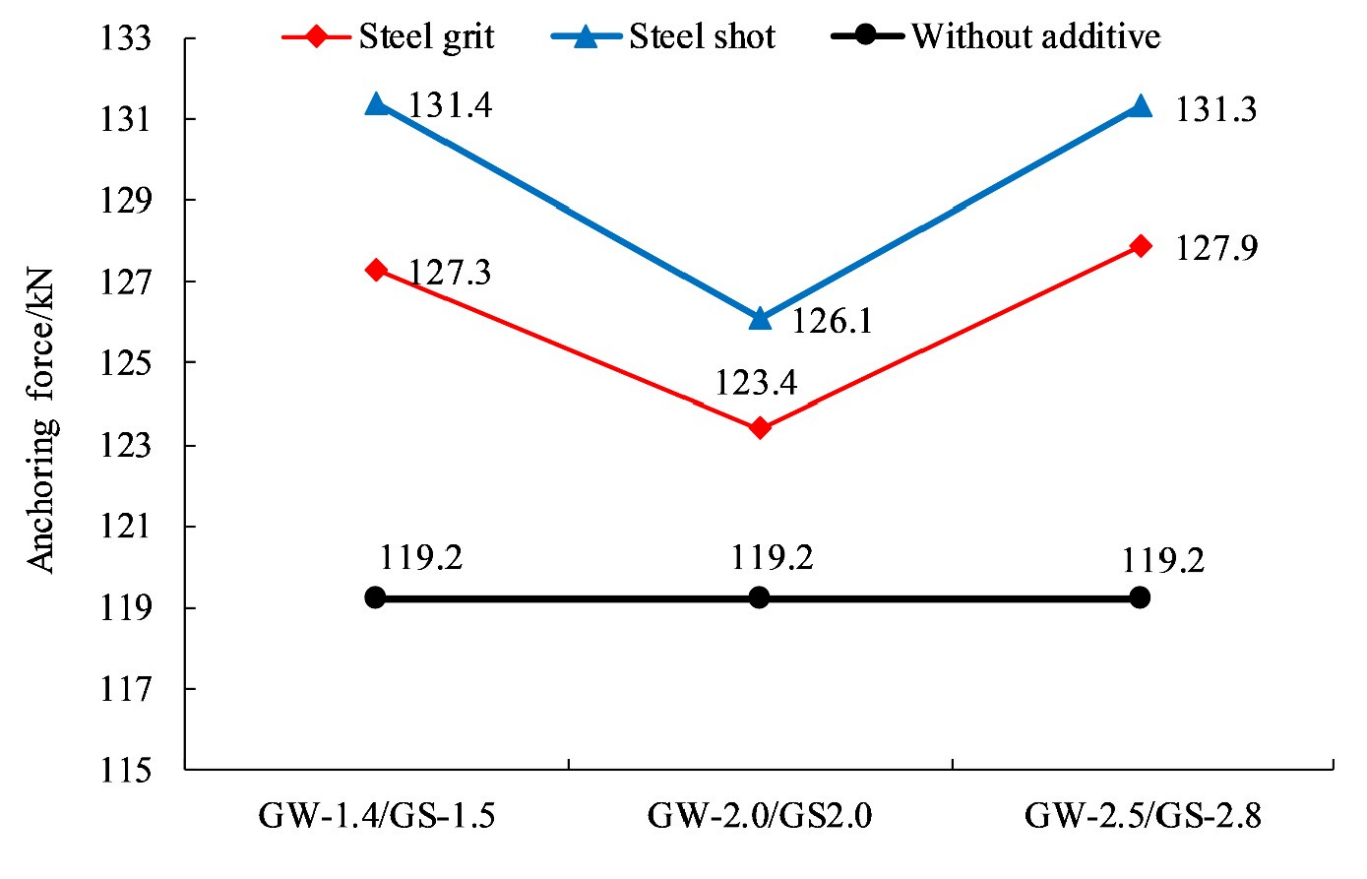
Effect of aggregate shape on anchoring force.

**Fig 22:**
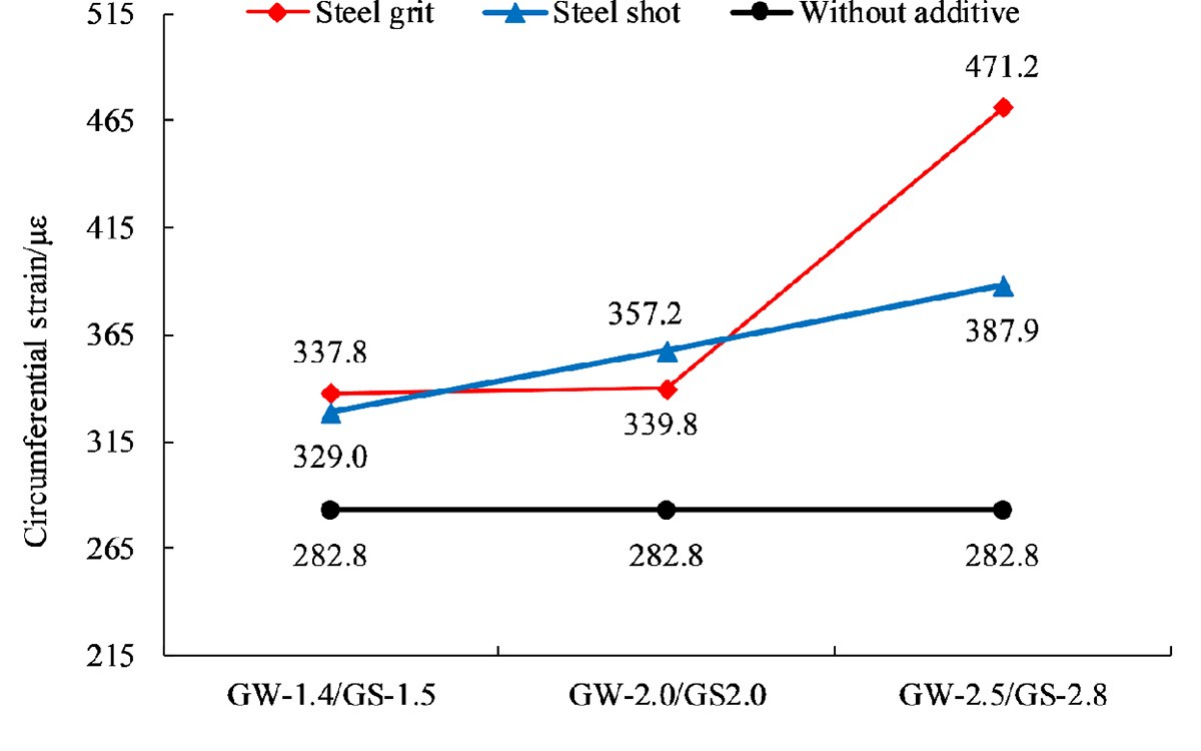
Effect of aggregate shape on circumferential strain.

**Fig 23:**
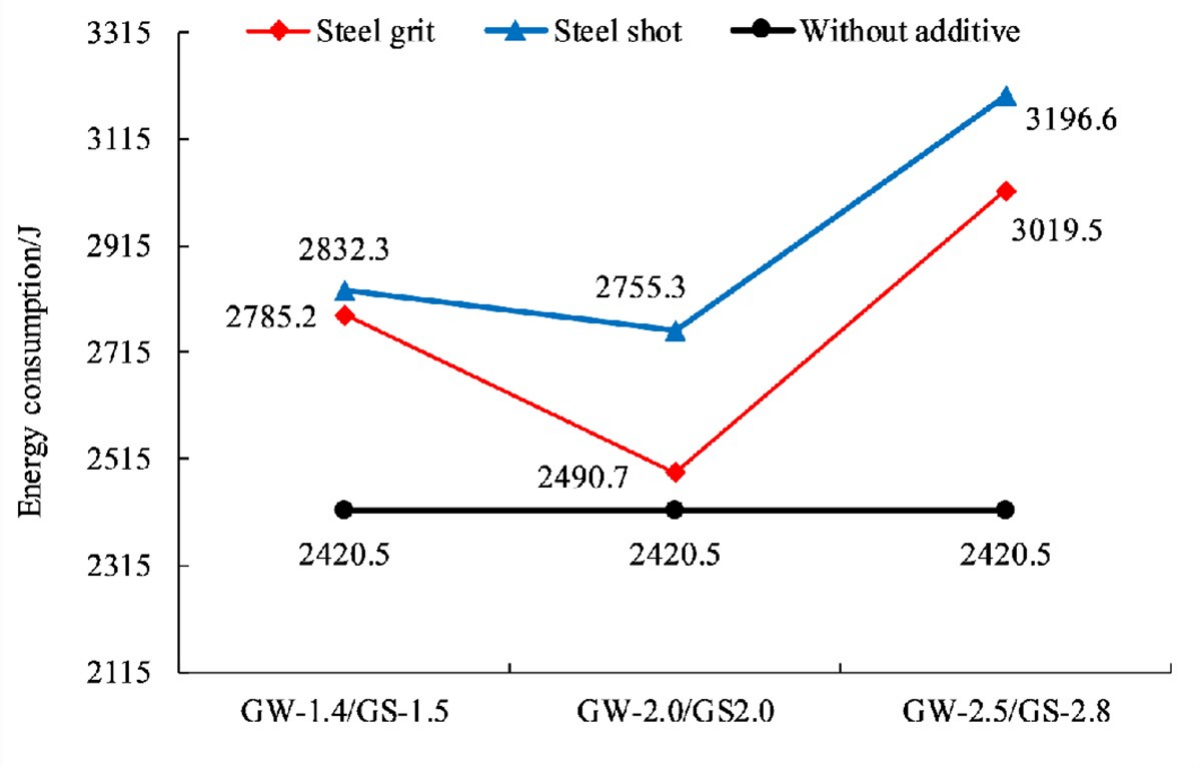
Effect of aggregate shape on energy consumption.

Figs 21–23 show the effects of two aggregate shapes on anchoring force, circumferential strain and energy consumption, ignoring the quantity but considering the size of aggregates. The results show that adding steel shots into resin anchoring agents is more helpful to improve anchorage performance and steel shots are better than steel grits in improving anchorage performance, but there is not much difference.

### Discussion on anchorage failure interface

**Fig 24:**
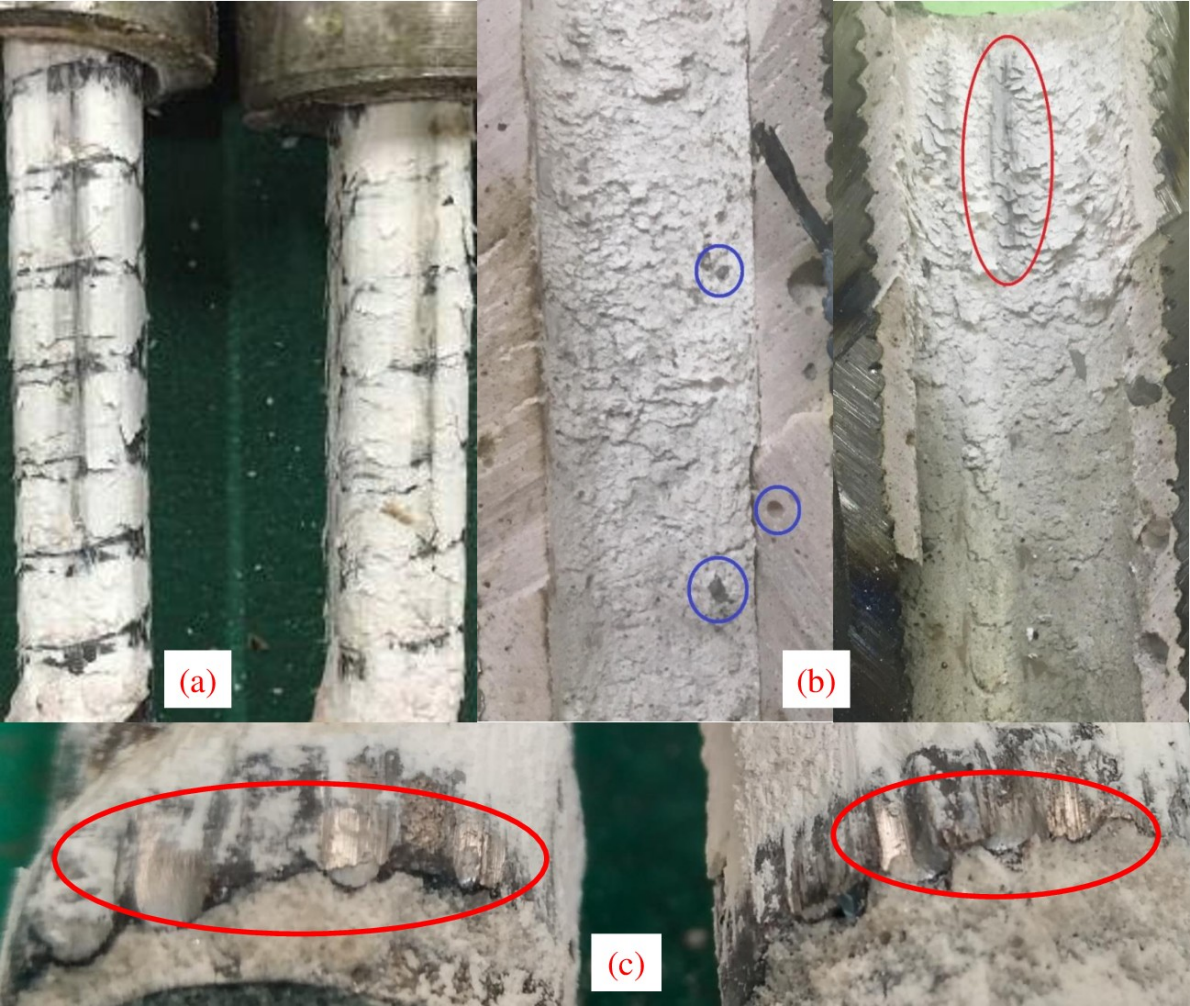
Failure surface observation (a) The scratches caused by the aggregates on the failure interface. (b) The scratch in the red circle is caused by aggregates, however, the aggregates in the blue circles do not produce a doweling effect. (c) The damage to the bolt ribs because of the doweling effect.

With the increase of axial force, the resin is subject to increased shear stress, which gradually causes interface debonding. Through observation of post-test specimens, it is found that the failure mode in this experiment is mainly shear failure of the resin, which is consistent with the conclusions of aforementioned literature [32–34]. However, the shearing deformation of the resin is different to the rough surface slippage typically occurred in geo-materials [39–41]. Fig 24(A) shows the scratches caused by the steel aggregates on the failure interface. Fig 24(B) shows the middle section of the steel sleeve after the experiment; the sawn in red circle is caused by scraping of the steel aggregates against surrounding resin during the pullout test, which affects the debonding interface geometry of the anchoring specimen. The average thickness of the resin annulus is 5.0 mm and the maximum steel aggregate size is 2.8 mm. Thus, some aggregates do not produce a doweling effect in the pullout procedure, such as the steel aggregates in the blue circles. Fig 24(C) shows the damage to the bolt ribs caused by passing through steel aggregates, which suggests that multiple steel aggregates produce a doweling affect at the failure interface.

According to anchorage failure analysis and observations of this study, it can be concluded that the shear resistance of the anchoring agent with steel aggregates while a bolting specimen is subject to axial load is mainly composed of following four components:

1. The initial confining pressure of the bolt generated during consolidation of grouting materials;
2. Cohesive strength of the resin;
3. Internal friction at slipping surface;
4. The doweling effect of the aggregates at the failure surface.

It should be noted that the anchorage failure is also related to the loading conditions, such as circular loading, creeping or unloading, the corresponding mechanisms may be different static axial loading [42]. Another important issue related to deformational behaviour of the resin is crack or fracture of the anchorage agent and surrounding [43–45]. The strength of the anchorage agent with or without steel particles is an important factor for the bolting performance.

### Discussion on the strength of anchoring agents

Rock bolting failure is closely related to anchorage agent failure. Therefore, increasing resin strength is an effective means to improve anchoring performance. The mechanical strength indicators commonly used for anchoring materials are mainly compressive strength and shear strength. Among them, uniaxial compressive strength (UCS) is the most widely used parameter, and it is also a standard for grouting agent quality. In geotechnical engineering research, Zhou et al found that the modulus of elasticity increased greatly, but the UCS decreased by about 10% when steel shots were added into the grouting [46]. Kilic et al studied the effect of aggregates on the strength of high-strength silica concrete, and test results showed that even if concrete with higher UCS aggregates is added, the UCS of the mixture will be limited by the bond strength, i.e., the concrete UCS does not change significantly with or without aggregates [47]. In the study of mortar grouting materials, Benmokrane et al performed pull-out tests on rebar anchored by six types of cement grouting. The results showed that the correlation between the UCS of the grouting material and the bond peak strength is extremely weak [48]. Wang and Li et al [49,50] conducted a series of triaxial tests on EPS-cement lightweight clay, and found that increasing EPS bead content will also reduce the effective elastic modulus and compressive strength of this lightweight bulk filling material. However, these conclusions are at odds with the results of some studies [22–25].

In this study, the compressive and shear strengths of the resin with and without steel aggregates were compared. It is found that the compressive and shear strengths and cohesion of the material improved to some extent, but the differences between steel grits and steel shots are not significant. However, for the pullout of rockbolts, the failure mode of the anchoring specimen is mainly shear failure of the resin; UCS is less important than shear strength of the grouting agent, especially in roadways where surrounding rock stress reaches medium to high levels. Therefore, the shear strength is an extremely reliable and important mechanical parameter of grouting agents for rockbolt support.

## Conclusions

This experimental study quantitatively analyzes the effects of the shape, size and quantity of steel aggregates on anchoring force, energy consumption and circumferential strain of the sleeve via pullout testing. The results show that:

When steel grits and shots are added, the compressive and shear strengths of resin improve by 8.4% and 11.8%, and 17.0% and 13.4%, respectively.

The increment of the average ultimate anchoring force, energy consumption and circumferential strain of the sleeve with aggregate quantities of 30, 40 and 50 is 7.9%, 7.5% and 6.5%, 19.2%, 15.0% and 18.6%, and 28.4%, 25.1% and 39.5%, respectively. The increase in the anchorage force is less significant than that of the energy consumption and the circumferential strain of the sleeve.

Neglecting the shape and size of the aggregates, the relationships of the anchoring force, energy consumption and circumferential strain of the sleeve with aggregate quantity can be expressed as Eqs (1)–(3), respectively. The increment of the average ultimate anchoring force, energy consumption and circumferential strain of the sleeve with steel grit quantities of 30, 40 and 50 is 7.9%, 7.5% and 6.5%; 19.2%, 15.0% and 18.6%, and 28.4%, 25.1% and 39.5%, respectively. The quantity of the steel aggregates has significant impact on anchoring performance.

Neglecting the shape and quantity of the steel aggregates, the relationships of the anchoring force, energy consumption and circumferential strain of the sleeve with aggregate size can be expressed as Eqs (4)–(6), respectively. The increment of the average ultimate anchoring force, energy consumption and circumferential strain of the sleeve with aggregate sizes (diameters) of 1.4~1.5, 2.0~2.0 and 2.5~2.8 mm are 8.5%, 4.6% and 8.7%, 16.0%, 8.4% and 28.4%, and 17.9%, 23.3% and 51.9%, respectively. The aggregate size (diameter) has significant impact on anchoring performance.

Even though aggregate shape affects anchorage performance of the bolting specimen, there is no significant difference between steel grits and steel shots.

From the above, by applying the doweling effect in the design of anchoring agents, it can be deduced that without changing the basic structure and composition of traditional anchoring agents, anchorage performance can be significantly improved through the addition of hard fine aggregates.

## Supporting information

S1 Fig The load-displacement curves of the specimens with steel grits. (TIF)

S2 Fig The load-displacement curves of the pullout specimens with steel shots. (TIF)
