# Supplementary figures and images for: Experimental study on anchorage performance of rockbolts by adding steel aggregates into resin anchoring agents
Source: PLoS One. 2021 Jul 28;16(7):e0255046. doi: 10.1371/journal.pone.0255046 (PMC8318225; doi:10.1371/journal.pone.0255046)

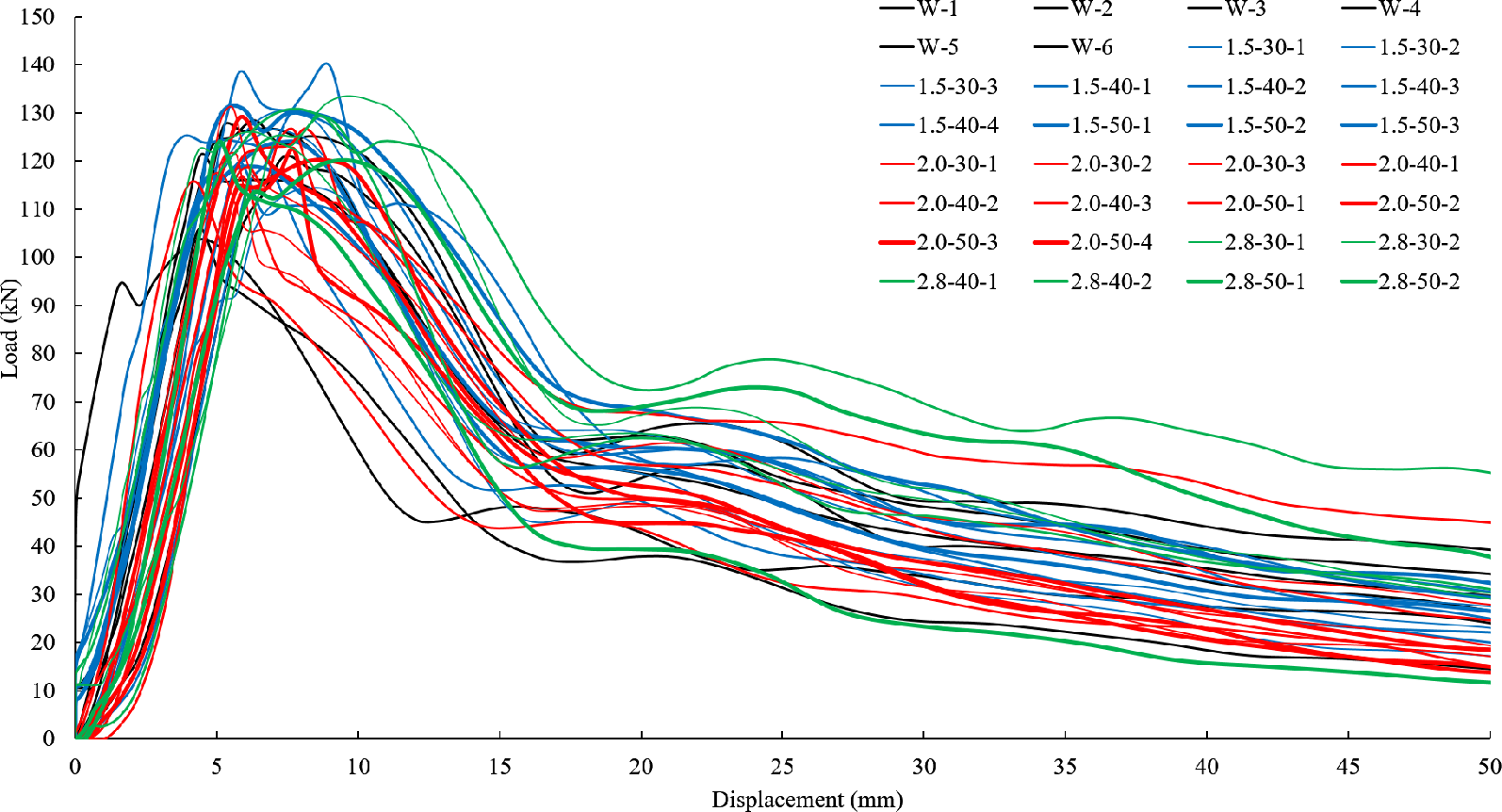

Supplement: S1 Fig — (TIF) [file pone.0255046.s001.tif]

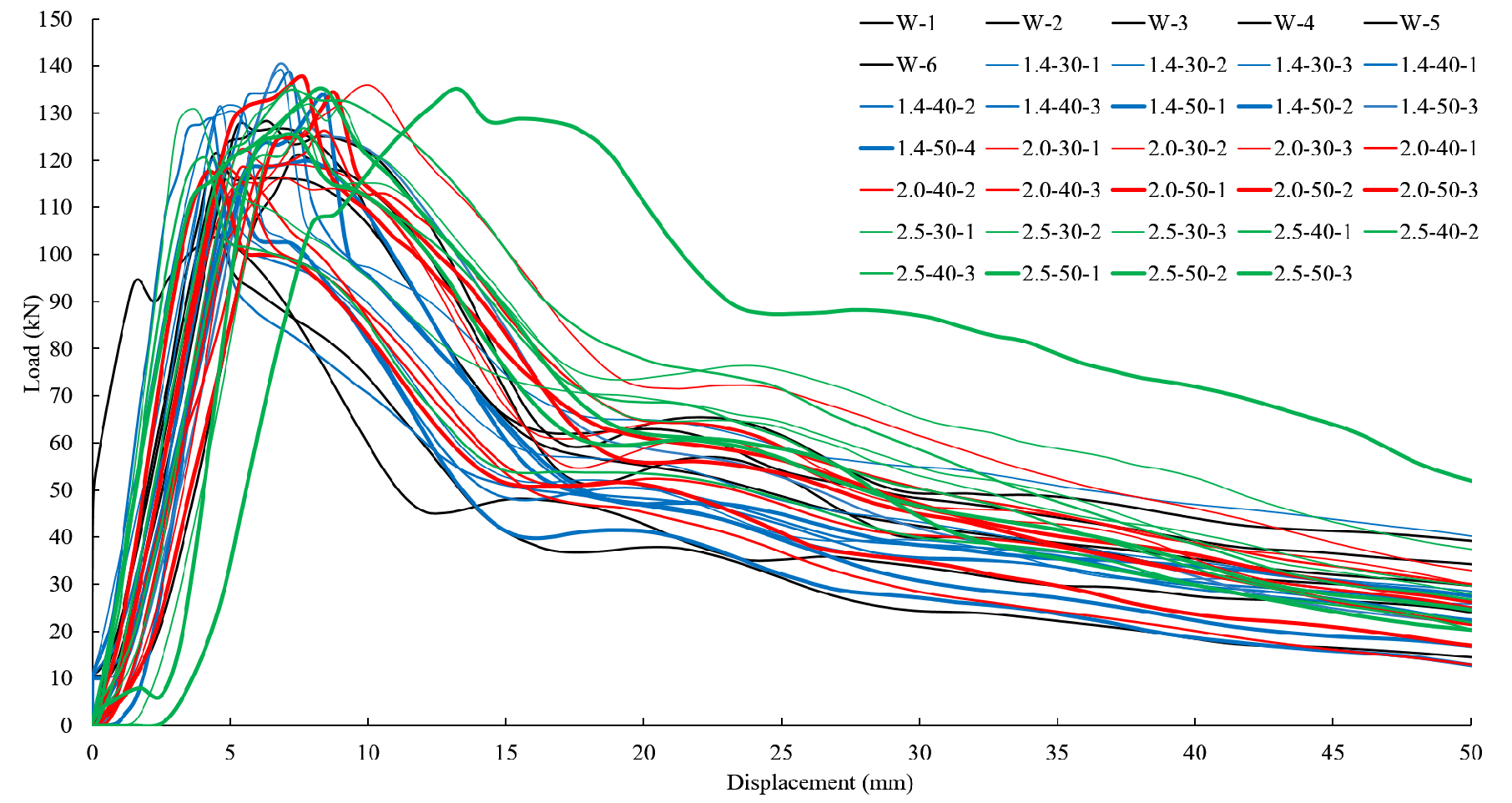

Supplement: S2 Fig — (TIF) [file pone.0255046.s002.tif]
